# Zika virus infection as a cause of congenital brain abnormalities and Guillain-Barré syndrome: A living systematic review

**DOI:** 10.12688/f1000research.19918.1

**Published:** 2019-08-14

**Authors:** Michel Jacques Counotte, Kaspar Walter Meili, Katayoun Taghavi, Guilherme Calvet, James Sejvar, Nicola Low

**Affiliations:** 1 Institute of Social and Preventive Medicine, University Bern, Bern, Switzerland; 2 Acute Febrile Illnesses Laboratory, Evandro Chagas National Institute of Infectious Diseases, Oswaldo Cruz Foundation, Rio de Janeiro, Rio de Janeiro, Brazil; 3 Division of High-Consequence Pathogens and Pathology, National Center for Emerging and Zoonotic Infectious Diseases, Centers for Disease Control and Prevention, Atlanta, GA, USA

**Keywords:** Zika, Disease outbreaks, arboviruses, causality, Guillain-Barré syndrome, congenital abnormalities

## Abstract

**Background:** The Zika virus (ZIKV) caused a large outbreak in the Americas leading to the declaration of a Public Health Emergency of International Concern in February 2016. A causal relation between infection and adverse congenital outcomes such as microcephaly was declared by the World Health Organization (WHO) informed by a systematic review structured according to a framework of ten dimensions of causality, based on the work of Bradford Hill. Subsequently, the evidence has continued to accumulate, which we incorporate in regular updates of the original work, rendering it a living systematic review.

**Methods:** We present an update of our living systematic review on the causal relation between ZIKV infection and adverse congenital outcomes and between ZIKV and GBS for four dimensions of causality: strength of association, dose-response, specificity, and consistency. We assess the evidence published between January 18, 2017 and July 1, 2019.

**Results:** We found that the strength of association between ZIKV infection and adverse outcomes from case-control studies differs according to whether exposure to ZIKV is assessed in the mother (OR 3.8, 95% CI: 1.7-8.7, I ^2^=19.8%) or the foetus/infant (OR 37.4, 95% CI: 11.0-127.1, I ^2^=0%). In cohort studies, the risk of congenital abnormalities was 3.5 times higher after ZIKV infection (95% CI: 0.9-13.5, I ^2^=0%). The strength of association between ZIKV infection and GBS was higher in studies that enrolled controls from hospital (OR: 55.8, 95% CI: 17.2-181.7, I ^2^=0%) than in studies that enrolled controls at random from the same community or household (OR: 2.0, 95% CI: 0.8-5.4, I ^2^=74.6%). In case-control studies, selection of controls from hospitals could have biased results.

**Conclusions:** The conclusions that ZIKV infection causes adverse congenital outcomes and GBS are reinforced with the evidence published between January 18, 2017 and July 1, 2019.

## Introduction

The Zika virus (ZIKV), a mosquito-borne flavivirus, caused a large outbreak of infection in humans in the Americas between 2015–2017 ( WHO Zika -Epidemiological Update). Since then, the circulation of ZIKV has decreased substantially in the Americas ^1^ but ZIKV transmission will likely continue at a lower level ^2^. Smaller outbreaks have been reported from countries in Africa and Asia, including Angola, India ^3^, and Singapore ^4^. Regions with endemic circulation, such as Thailand ^5^, have the potential for new ZIKV outbreaks with adverse outcomes ^6^.

The World Health Organization (WHO) declared ZIKV as a cause of adverse congenital outcomes and Guillain-Barré syndrome (GBS) as early as September 2016 ^7^, informed by a systematic review of evidence structured according to a framework of ten dimensions of causality, based on Bradford Hill ( Table 1) ^8^. The accumulation of evidence on the adverse clinical outcomes of ZIKV has barely slowed down since the WHO declared the Public Health Emergency of International Concern (PHEIC) on February 1 ^st^, 2016, with approximately 250 research publications on ZIKV appearing every month (see Zika Open Access Project). We updated the systematic review to January 18, 2017 as a living systematic review by introducing automated search methods to produce a high quality, up to date, online summary of research ^9^ about ZIKV and its clinical consequences, for all the causality dimensions ^10^.

**Table 1.** Comparison of the search strategy included study designs and causality dimensions addressed in the different review periods. The latest and previous versions of this table are available as extended data ^16^.

Review	Baseline ^8^	Update 1 ^10^	Update 2 [this review]
**Period**	<May 30, 2016	May 30, 2016-January 18, 2017	>January 18, 2017 -July 01, 2019
**Search strategy**	“ZIKV” or “Zika”	“ZIKV” or “Zika”	Focussed search strategy (Supplementary File 2)
**Study design**	Epidemiological studies; *in vivo*/ *in vitro* studies; surveillance reports		Epidemiological studies
**Dimensions of the causality** framework based on Bradford Hill *	•Temporality (cause precedes effect)		
	•Biological plausibility of proposed biological mechanisms		
	•Strength of association		•Strength of association
	•Exclusion of alternative explanations		
	•Cessation (reversal of an effect by experimental removal of, or observed a decline in, the exposure)		
	•Dose-response relationship		•Dose-response relationship
	•Experimental evidence from animal studies		
	•Analogous cause-and-effect relationships found in other diseases		
	•Specificity of the effect		•Specificity of the effect
	•Consistency of findings across different study types, populations and times		•Consistency of findings across different study types, populations and times

* The causality framework is described elsewhere in detail:

[ https://f1000researchdata.s3.amazonaws.com/supplementary/13704/95380c74-7569-4049-bf3b-b2832794bdf9.docx].

Since 2017, understanding about the pathogenesis of how ZIKV causes congenital abnormalities has evolved ^11, 12^. The quality of diagnostic methods, especially for acute ZIKV infection, has also improved ^13– 15^. More importantly, understanding of the limitations of diagnostic testing, and the need for interpretation in the context of other flavivirus infections, has developed. Important epidemiological questions about the associations between ZIKV infection and adverse congenital outcomes and GBS remain unanswered, however. Much of the early epidemiological evidence, which relied on surveillance data, was limited in use because of issues with the quality of the reporting and case definitions. The reported strength of association between ZIKV and adverse outcomes has varied in studies of different designs and in different settings. Evidence for a dose-response relationship with higher levels of exposure to ZIKV resulting in more severe outcomes, of clinical findings that are specific to ZIKV infection, or of adverse outcomes caused by different lineages of ZIKV was not found in the earlier systematic reviews.

The objective of this study is to update epidemiological evidence about associations between ZIKV infection and adverse congenital outcomes and between ZIKV and GBS for four dimensions of causality: strength of association, dose-response, specificity, and consistency.

## Methods

We performed a living systematic review, which we have described previously ^10^. This review updates the findings of the previous reviews ^8, 10^ and will be kept up to date, in accordance with the methods described below. Reporting of the results follows the Preferred Reporting Items of Systematic reviews and Meta-Analyses (PRISMA) statement (Extended data, Supplementary File 1 ^17^) ^18^.

### Focus on epidemiological aspects of causality

This review and subsequent updates will focuse on four dimensions of causality that are examined in epidemiological study designs: strength of association, dose-response relationship and specificity of effects and consistency of association (Extended data, Table 1, Supplementary File 2 ^17^). Evidence for domains of causality that are typically investigated in *in vitro* and *in vivo* laboratory studies ( Table 1) was not sought. In the absence of licensed vaccines or treatments for ZIKV infection, we did not search for evidence on the effects of experimental removal of ZIKV.

### Eligibility criteria

**Figure 1.**
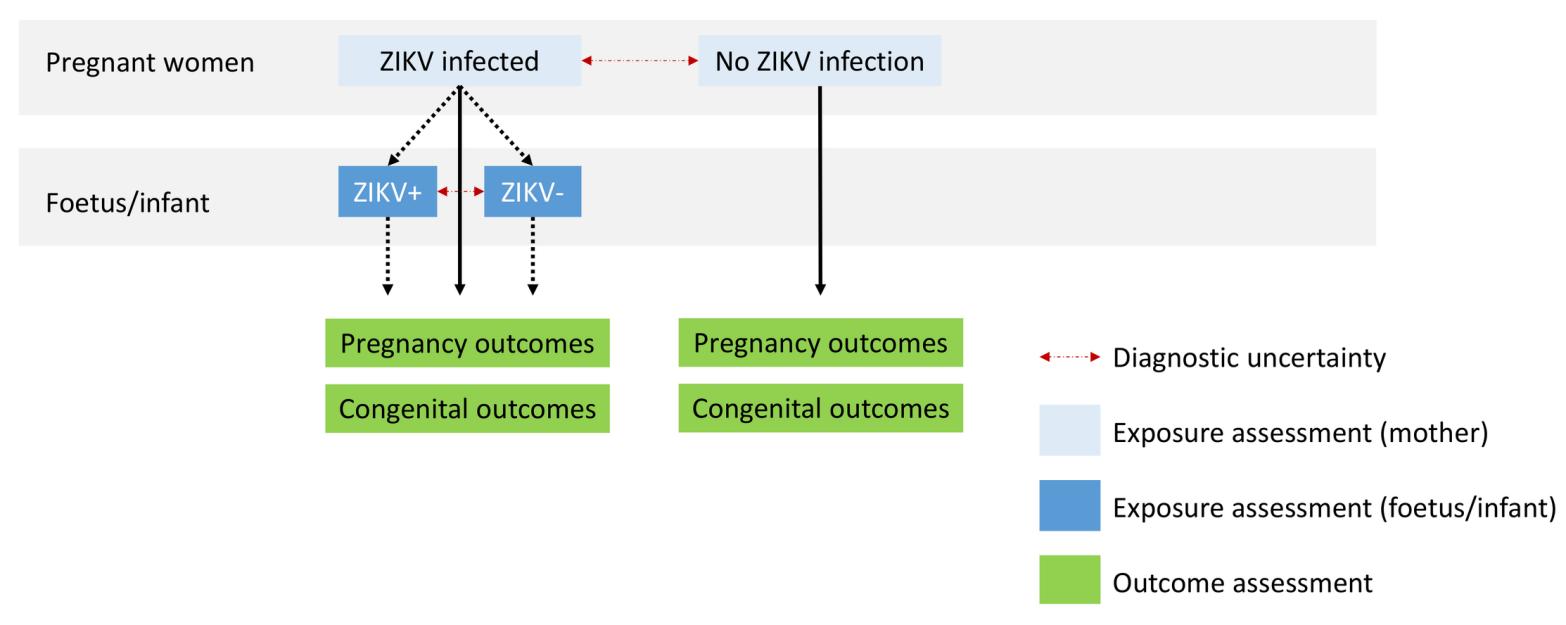
For congenital abnormalities due to ZIKV, exposure assessment in mother-infant pairs can be performed in the mother or the foetus or infant. The latest and previous versions of this figure are available as extended data ^16^.

We considered epidemiological studies that reported original data and assessed ZIKV as the exposure and congenital abnormalities or GBS as the outcomes. We based the exposure and outcome assessment on the definitions used in the publications. We applied the following specific inclusion criteria (Extended data, Supplementary File 2 ^17^):

Strength of association: at the individual level, we selected studies that included participants both with and without exposure to ZIKV ( Figure 1), such as cohort studies and case-control studies. At the population level, we included studies that assessed the outcome during the ZIKV outbreak and provided a comparison with pre or post-outbreak incidence of the outcome.

Dose-response relationship: we included studies that assessed the relation between the level of the viral titre or the presence or severity of the symptoms and the occurrence or severity of the outcome.

Specificity of the outcome for ZIKV exposure: we included studies that assessed whether the pathological findings in cases with the outcome are specific for ZIKV infection.

Consistency: we looked at eligible studies to determine the consistency of the relationship between ZIKV exposure and the outcomes across populations, study designs, regions or strains.

### Search and information sources

We searched PubMed, Embase, LILACS and databases and websites of defined health agencies (Extended data, Supplementary File 2 ^16^). We included search terms for the exposure, the outcome and specific study designs. We also performed searches of the reference lists of included publications. A detailed search strategy is presented in Supplementary File 2. For this review, the search covered the period from January 19, 2017 to July 1, 2019.

### Study selection and extraction

One reviewer screened titles and abstracts of retrieved publications. If retained, the same reviewer screened the full text for inclusion. A second reviewer verified decisions. One reviewer extracted data from included publications into piloted extraction forms in REDCap (version 8.1.8 LTS, Research Electronic Data Capture) ^19^. A second reviewer verified data entry. Conflicts were resolved by consulting a third reviewer.

### Synthesis of evidence

First, we summarised findings for each dimension of causality and for each outcome descriptively. Where available, we calculated unadjusted odds ratios (OR) or risk ratios (RR) and their 95% confidence interval (CI) from published data for unmatched study designs. For matched study designs, we used the effect measure and 95% CI presented by the authors. For publications that presented results for multiple measures of exposure and/or outcome, we compared these results. We applied the standard continuity correction of 0.5 for zero values in any cell in the two-by-two table ^20^. We used the I² statistic to describe the percentage of variation across studies that is due to heterogeneity for reasons other than chance ^21^. Quantitative synthesis was performed using R 3.5.1 ^22^. We conducted random effects meta-analyses using the R package metafor (version 2.0-0) ^20^. Finally, we compared descriptive and quantitative findings from this review period with previous versions of the review ^8, 10^.

### Searching and screening frequency

Daily searches of PubMed, Embase and LILACS are automated and monthly searches are performed manually for other information sources in the first week of the month (Extended data, Supplementary File 2 ^17^), with screening of all retrieved publications on the same day. The search strategy consisted of a combination of free terms and MESH terms that identified the exposure and outcomes (Extended data, Supplementary File 2 ^17^). Searches from multiple sources were combined and automatically deduplicated by an algorithm that was tested against manual deduplication. Unique records enter a central database, and reviewers are notified of new content.

### Frequency of results update

The tables and figures presented in this paper will be updated every six months as a new version of this publication. As soon as new studies are included, their basic study characteristics are extracted and provided online https://zika.ispm.unibe.ch/assets/data/pub/causalityMap/.

### Duration of maintenance of the living systematic review

We will keep the living systematic review up to date for as long as new relevant data are published and at least until October 31, 2021, the end date of the project funding.

### Risk of bias/Certainty of evidence assessment

To assess the risk of bias of cohort studies and case-control studies, we compiled a list of questions in the domains of selection bias, information bias, and confounding, based on the quality appraisal checklist of the United Kingdom National Institute for Health and Care Excellence (NICE) and literature ^23^. Two independent reviewers conducted the quality assessment. Disagreements were resolved by a third reviewer.

## Results

### Search results from January 19, 2017 to July 1, 2019 (Update 2)

From January 19, 2017 to July 1, 2019 we screened 1941 publications, of which we included 638 based on title and abstract. After reviewing the full text, 249 publications were included ( Table 2, Figure 3). Of these publications, 195 reported on congenital abnormalities linked to ZIKV ^24– 217^ and 59 on GBS ^4, 44, 118, 201, 203, 206, 218– 270^. Five outbreak reports described both outcomes ^44, 118, 201, 203, 206^.

**Table 2.** Included publications in the baseline review, update 1 and update 2 (this version), by outcome and epidemiological study design. The latest and previous versions of this table are available as extended data ^16^.

Outcome	Adverse congenital outcomes, number of publications			GBS, number of publications		
Review period/version	Baseline *	Update1 ^†^	Update2	Baseline *	Update1 ^†^	Update2
**Study design**						
Case report	9	13	39	9	5	17
Case series	22	12	62	5	11	22
Case-control study	0	2	10	1	1	7
Cohort study	1	8	35	0	0	0
Cross-sectional study	2	1	19	0	1	3
Controlled trials	0	0	0	0	0	0
Ecological study/outbreak report	5	4	27	19	7	9
Modelling study	2	0	3	0	0	1
**Total:**	**41**	**40**	**195**	**34**	**25**	**59**

* Baseline review, earliest date of each information source to May 30, 2016 ^8^;

† Update 1, May 30, 2016 to January 18, 2017 ^10^.

**Figure 2.**
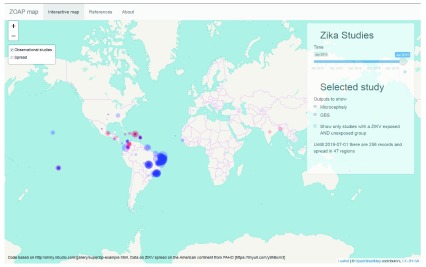
Map of the epidemiological studies that report on adverse congenital outcomes (blue) or Guillain-Barré syndrome (red) associated with Zika virus exposure. The size of the points correspond with the number of exposed individuals with the adverse outcome, according to the definitions used in the publications. The latest and previous versions of this figure are available as extended data ^16^.

**Figure 3.**
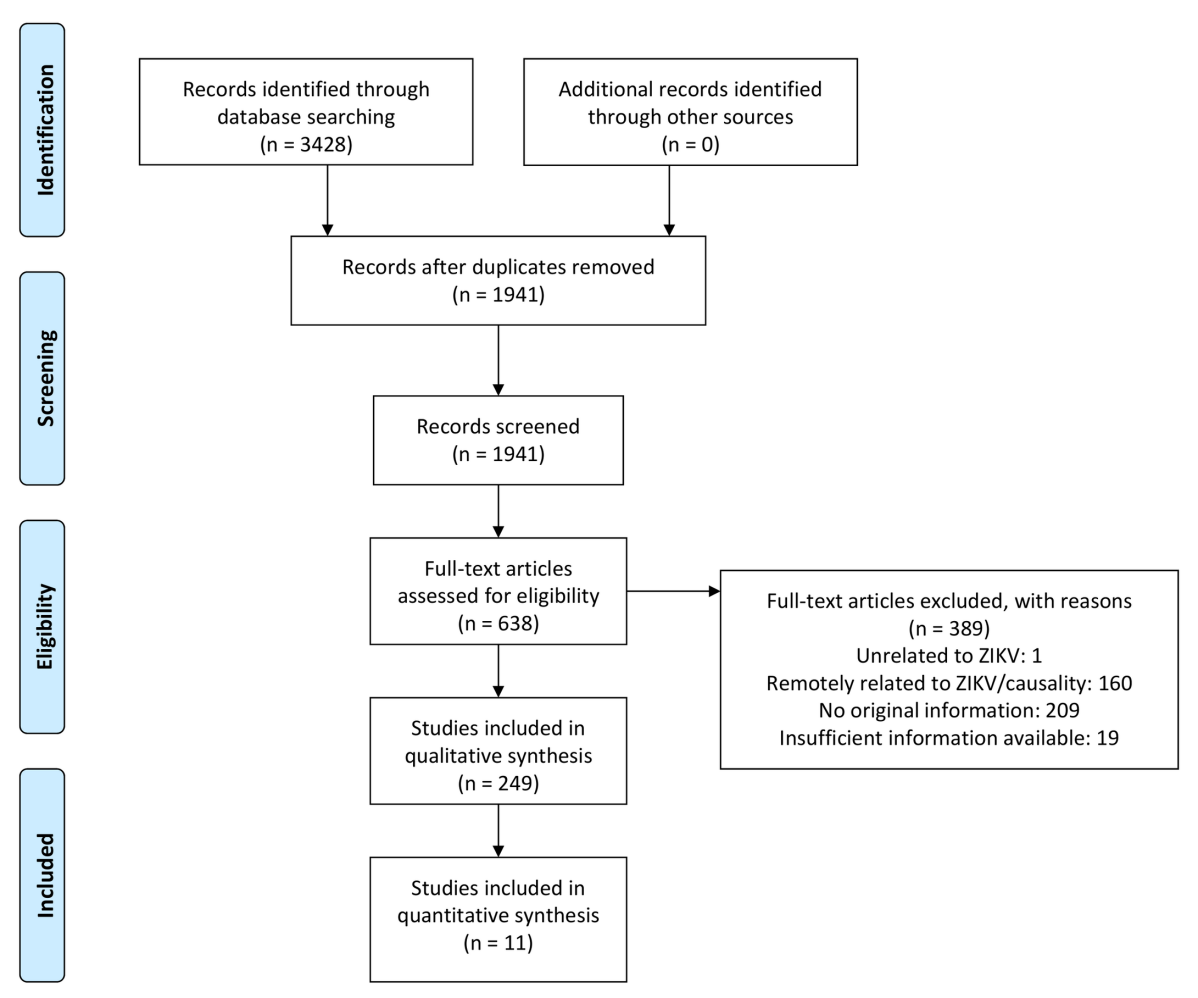
PRISMA flow-chart of publications retrieved, screened and included between January 18, 2017 and July 1, 2019. Adapted from: Moher *et al.* (2009) ^18^. The latest and previous versions of this figure are available as extended data ^16^.

### Adverse congenital outcomes

We included 39 case reports ^24– 61^, 62 case series ^62– 123^, 10 case-control studies ^124– 133^, 35 cohort studies ^134– 168^, 19 cross-sectional studies ^169– 187^, seven ecological studies ^188– 194^, three modelling studies ^195– 197^ and 20 outbreak reports ^198– 217^ that report on congenital abnormalities linked to ZIKV.

***Causality dimensions***

***Strength of association.*** **Individual level:** In this review period, five case-control studies reported on strength of association, four in Brazil (n=670 participants) ^125, 126, 128, 130^ and one in French Polynesia (n=123 participants) ^131^. The studies assess adverse pregnancy outcomes including infants born with microcephaly, according to exposure to ZIKV for cases. Of these, all studies matched controls, based on gestational age and/or region. During the review period up to January 18, 2017, we included one case-control study ^271^, which we replaced with a publication reporting the final results of the study ^126^. The meta-analyses incorporate estimates from studies identified in all review periods.

Assessment of exposure status varied between the studies (Extended data, Supplementary File 3 ^17^). In five case-control studies, exposure to ZIKV was assessed in the mother, based on clinical symptoms of ‘suspected Zika virus infection’ ^125^, or presence of maternal antibodies measured by IgM (Kumar *et al.* (2016) ^272^), PRNT (de Araujo *et al.* (2018) ^126^, Subissi *et al.* (2018) ^131^), or both PRNT and IgG (Moreira-Soto *et al.* (2018)) maternal antibody ^127^.

**Figure 4.**
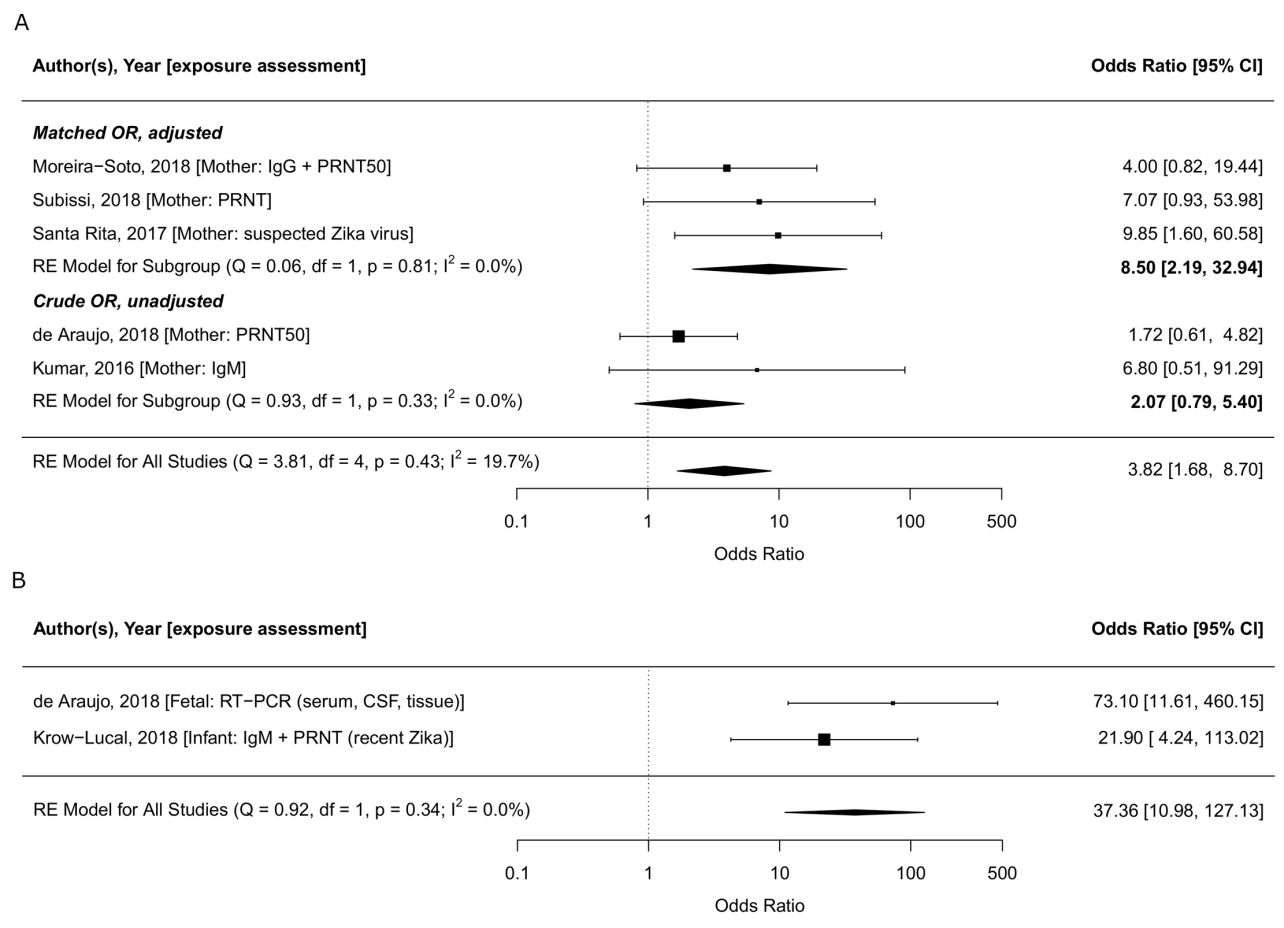
Forest plot and meta-analysis of case-control studies reporting on ZIKV infection assessed in mothers ( **A**) and in infants ( **B**) and adverse congenital outcomes (microcephaly, congenital malformations, central nervous system abnormalities). The odds ratio from the five case-control studies that assess exposure in mothers combined is 3.8 (95% CI: 1.7-8.7, tau ^2^=2.37, I ^2^=19.8%); the odds ratio for the studies that assess exposure in infants is 37.4 (95% CI: 11.0-127.1, tau ^2^=0, I ^2^=0%). The odds ratios are plotted on the log scale. Abbreviations: CSF, cerebrospinal fluid, PRNT, plaque reduction neutralisation test; RE, random effects; RT-PCR, reverse transcription polymerase chain reaction. The latest and previous versions of this figure are available as extended data ^16^.

In meta-analysis, we found that the odds of adverse congenital outcomes (microcephaly or congenital abnormalities) were 3.8 times higher in ZIKV-infected mothers (95% CI: 1.7-8.7, tau ^2^=0.18, I ^2^=19.8%, Figure 4). Moreira-Soto *et al.* (2018) found that in Bahia, Brazil, Chikungunya infection was also associated with being a case ^127^.

In two matched case-control studies, exposure to ZIKV was assessed in infants; Araujo *et al.* found a 73.1 (95% CI 13·0–Inf) times higher odds was reported for microcephaly when ZIKV infection was assessed by reverse transcription polymerase chain reaction (RT-PCR) in the neonate ^126^. Krow-Lucal *et al.* (2018) found an OR of 21.9 (95% CI: 7.0-109.3) based on evidence of recent Zika infection assessed using IgM followed by PRNT in infants in Paraiba, Brazil ^128^. When exposure was assessed at the infant-level, the combined odds of adverse congenital outcomes was 37.4 times higher (95% CI: 11.0-127.1, tau ^2^=0, I ^2^=0%, Figure 4).

**Figure 5.**
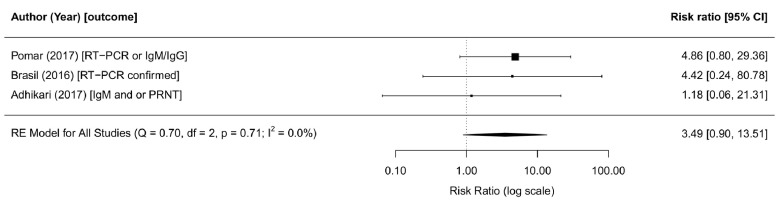
Forest plot and meta-analysis of cohort studies reporting on ZIKV infection and adverse congenital outcomes. The risk ratio from the random effects model is 3.5 (95% CI: 0.9-13.5, tau2=0,I ^2^=0%). The risk ratios are provided on the log scale. Abbreviations: ZIKV, Zika virus; PRNT, plaque reduction neutralisation test; RE, random effects; RT-PCR, Reverse transcription polymerase chain reaction. The latest and previous versions of this figure are available as extended data.

In this review period, one cohort study reported on strength of association, in 610 pregnant women returning from ZIKV-affected areas in Central and South America to the USA ^138^. Maternal ZIKV exposure was measured using RT-PCR or IgM followed by plaque reduction neutralisation test (PRNT). Among the 28 infants born to ZIKV-infected mothers, none was diagnosed with microcephaly and, one was born with a major malformation. In the ZIKV-unexposed group, eight out of 306 had major malformations. A complete overview of different outcomes assessed is presented in the extended data, Supplementary File 3 ^17^. During the review period up to January 18, 2017, we included two cohort studies, one in women with rash and fever (Brasil *et al.* (2016)) and one in unselected pregnant women (Pomar *et al.* (2017)) ^273, 274^. In meta-analysis of all three studies, we found that the risk of microcephaly was 3.5 times higher in ZIKV-infected mothers of babies (95% CI: 0.90-13.51, tau ^2^=0, I ^2^=0%, Figure 5).

**Population level:** At a population level, data from Mexico collected at different altitudes during the ZIKV outbreak, showed that the risk of microcephaly was increased in regions at altitudes below 2200m, in which ZIKV can circulate ^196^. Hay *et al.* (2018) reanalysed surveillance data from Colombia and northeast Brazil and concluded that time-dependent reporting changes might have caused apparent inconsistencies in the proportion of congenital abnormalities as a result of maternal ZIKV infection ^197^.

***Dose response.*** Halai *et al.* (2017) ^120^ examined the severity of congenital outcomes according to measures of the severity of maternal ZIKV infection in a subset of mothers in the cohort presented by Brasil *et al.* (2016) ^274^. They evaluated ZIKV load, assessed by RT-PCR using the cycle threshold (CT) as a measure of number of RNA copies, and a severity score of symptoms in 131 pregnant women. They concluded that neither higher viral load nor more severe symptoms was associated with more severe congenital abnormalities ^136^. Moreira-Soto *et al.* found higher maternal antibody titers in microcephaly cases compared with controls ^127^. In previous review periods, Honein *et al.* (2016) compared outcomes in neonates born to symptomatic and asymptomatic infected pregnant women returning to the USA with possible ZIKV infection and found no differences ^275^.

***Specificity.*** Although some outcomes, such as lingual phenotype ^177^ or neurogenic bladder ^276^, have been hypothesised as a specific phenotype for congenital ZIKV infection, no additional evidence was identified that certain congenital adverse findings are specific for congenital ZIKV infection.

***Consistency.*** **Geographical region**: All four WHO geographic regions (the Africa region [AFRO], the American region [AMRO], the South-East Asian region [SEARO] and the Western Pacific region [WPRO]) with past or active ZIKV transmission have now reported congenital abnormalities due to ZIKV infection. During this review period, the first congenital abnormality due to infection with the Asian lineage of the virus on the African mainland occurred in a traveller returning from Angola ^47^. Possible cases of congenital abnormalities have occurred in Guinea-Bissau ^96^. In the most recent WHO situation report from March 2017, two cases of microcephaly are documented in Thailand and one in Vietnam, which were also described in detail in other works ^24, 54, 107^. We identified another publication on congenital abnormalities due to endemic ZIKV in Cambodia ^110^. The occurrence of congenital adverse outcomes in AFRO, SEARO, and WPRO seems sporadic, despite the endemic circulation of ZIKV. As noted above, the observed complication rate varied strongly between regions. Extended data, Supplementary File 3 provides a full overview of the published studies on congenital abnormalities per region and country ^17^.

**Traveller/non-traveller populations:** In this update, we found further evidence that congenital abnormalities occurred in infants born to women travellers returning from ZIKV-affected areas and women remaining in those areas. In total, 25 publications report on 272 congenital abnormalities due to ZIKV infection in travellers ^27, 29, 30, 32, 34, 36, 38, 42, 45, 56, 58, 61, 89, 94, 98, 109, 117, 122, 123, 137, 140, 151, 153, 166, 173^, with 109 publications reporting congenital abnormalities due to ZIKV in 2652 non travellers ^24– 26, 28, 31, 33, 35, 37, 39– 41, 43, 44, 46– 48, 50, 51, 54, 57, 60, 62– 79, 81, 83– 85, 87, 88, 90– 93, 95– 97, 100, 101, 104– 106, 110– 113, 115, 116, 118, 119, 121, 124– 135, 142– 147, 149, 152, 154, 156– 164, 167, 168, 171, 172, 175– 180, 182, 186^

In this review period, evidence emerged that transmission through sexual contact with infected travellers also resulted in foetal infection ^58, 59^.

**Study designs:** The association between ZIKV infection and congenital abnormalities was consistent across different study designs ( Table 2).

**Lineages**: We found no new evidence of consistency across different lineages from observational studies. The currently observed adverse congenital outcomes are linked to the ZIKV of the Asian lineage.

### Risk of bias assessment

In all case-control studies, uncertainty about the exposure status due to imperfect tests could result in a bias towards the null. Some studies might suffer from recall bias where exposure was assessed by retrospectively asking about symptoms ^125, 131^. For the cohort studies ^138, 274^, the enrolment criteria were based on symptomatology. As a result, even in the absence of evidence of ZIKV, the unexposed groups might have had conditions that were unfavourable to their pregnancy. We expect this to bias the results towards the null or underestimate the true effect. Owing to imperfect diagnostic techniques, both false positives (IgM, cross reactivity) and false negatives (due to the limited detection window for RT-PCR) might occur, potentially resulting in bias; the direction of this bias would often be towards the null. None of the studies controlled for potential confounding. Extended data, Supplementary File 4 provides the full risk of bias assessment of the studies included in the meta-analysis ^17^.

### GBS

During this review period, we included 17 case reports ^44, 218– 233^, 22 case series ^118, 234– 254^, seven case-control studies ^4, 255– 260^, one ecological study ^264^, one modelling study ^265^ and eight outbreak reports ^201, 203, 206, 266– 270^ that reported on ZIKV infection and GBS.

During this review period, we included 17 case reports ^46, 220– 235^, 22 case series ^120, 236– 256^, seven case-control studies ^4, 257– 262^, one ecological study ^266^, one modelling study ^265^ and eight outbreak reports ^201, 203, 206, 266– 270^ that reported on ZIKV infection and GBS.

***Causality dimensions***

***Strength of association.*** **Individual level.** The number of studies reporting on the strength of association between ZIKV infection and GBS at an individual level increased substantially. We identified five case-control studies ^255– 259^ published since the previous update, which included one case-control study from French Polynesia ^277^. All studies were matched for age and place of residence. In the studies from Brazil, Colombia, Puerto Rico and New Caledonia, temporal clustering of cases in association with ZIKV circulation was documented ^255– 258^. In Bangladesh, ZIKV transmission was endemic ^259^. Exposure assessment was based on serology ^255, 256^ or a combination of RT-PCR and serology ^257– 259^. Extended data, Supplementary File 3 shows the variability in ORs according to criteria for ZIKV exposure assessment, based on unmatched crude data extracted from each case-control study ^17^.

**Figure 6.**
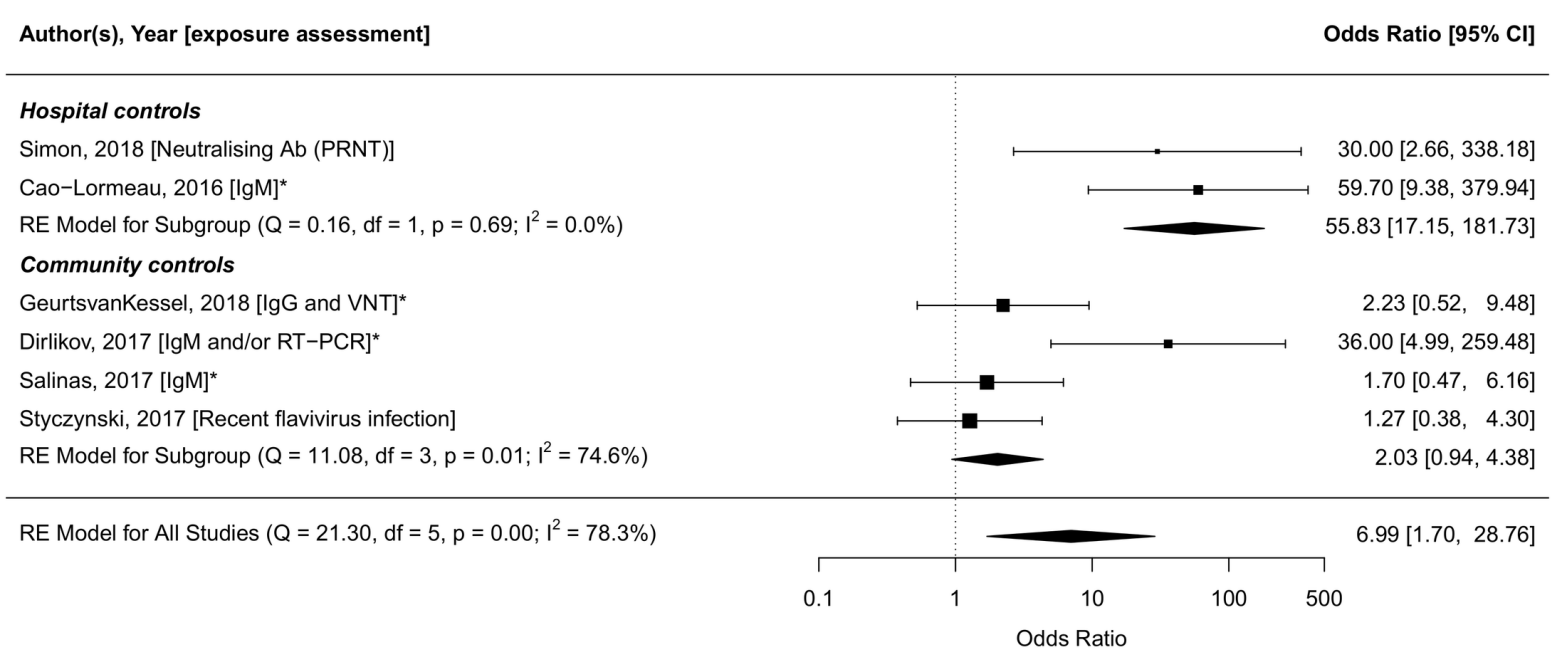
Forest plot of six included case-control studies and their exposure assessment. Odds ratios (ORs) are shown on the log-scale. The meta-analysis is stratified by the selection of controls: Hospital controls, or community/household controls. Most similar exposure assessment measures are compared (IgM ^256, 258, 259, 277^, recent flavivirus infection ^255^, or IgM and/or RT-PCR ^257^). OR: 7.0 [95% CI: 1.7-28.8, tau2=2.78, I ^2^=78.3%]. ORs from studies marked with an asterisk (*) are matched ORs, unmarked studies provided crude ORs. The latest and previous versions of this figure are available as extended data ^16^.

Figure 6 shows the association between GBS and ZIKV infection, using the diagnostic criteria that were most similar across studies. Heterogeneity was considerable (I ^2^=78.3%), but was reduced slightly after stratification based on the method of selection of controls. The summary OR was higher in studies that enrolled controls from hospital (OR: 55.8, 95% CI: 17.2-181.7, tau ^2^=0, I ^2^=0%) ^258, 277^ than in studies that enrolled controls at random from within the same community ^255– 257^ or from the same household ^259^ (OR: 2.0, 95% CI: 0.8-5.4, tau ^2^=0.46, I ^2^=74.6%). Amongst studies with community controls, ORs were lower when enrolment and assessment took place several months after onset of symptoms ^255, 256^ than in studies with contemporaneous enrolment ^257, 259^. To further illustrate the heterogeneity in exposure assessment between and within the studies, we provide additional aggregations of the data in Extended data, Supplementary File 3 ^17^.

***Population level.*** At a population level, Mier-Y-Teran-Romero *et al.* (2018) found that the estimated incidence of GBS ranged between 1.4 (0.4–2.5) and 2.2 (0.8–5.0) per 10,000 ZIKV infections comparing surveillance/reported cases from Brazil, Colombia, Dominican Republic, El Salvador, French, Honduras, Puerto Rico, Suriname, Venezuela, and Micronesia. The across-location minimum and maximum estimates were used to estimate an average risk of having GBS and being reported after ZIKV infection across locations of approximately 2.0 GBS cases per 10,000 infections (95% credible interval 0.5–4.5 per 10,000 ZIKV infections) ^265^.

***Dose response.*** In a case-control study, Lynch *et al.* (2018) found higher titres of neutralising antibodies in ZIKV-infected GBS cases than in patients with symptomatic ZIKV infection but without GBS ^260^.

***Specificity.*** Dirlikov *et al.* (2018) compared Puerto Rican GBS cases reported through public health surveillance that were preceded by ZIKV and cases that were not preceded by ZIKV infection ^249^. Clinical features involving cranial nerves were observed more frequently in ZIKV-related cases and, at a six-month follow-up visit, residual cranial neuropathy was noted more often in this group. However, clinical symptoms did not allow a distinction to be made between ZIKV and non-ZIKV related GBS.

***Consistency.*** **Geographical region**: During this review period, GBS likely due to ZIKV infection was reported in Asia; including Thailand, Bangladesh, Singapore and India ^4, 48, 252, 259^. Publication in the WHO Region of the Americas followed the pattern as observed before and no GBS linked to ZIKV infection was reported in Africa. Extended data, Supplementary File 3 provides a full overview of the published studies on congenital abnormalities by region and country ^17^. In a reanalysis of surveillance data from the Region of the Americas, Ikejezie *et al.* (2016) found consistent time trends between GBS incidence and ZIKV incidence ^264^.

**Traveller/non-traveller populations:** In studies included in this update, we found additional evidence of GBS in both travellers and non-travellers with ZIKV infection. Ten publications report on 11 travellers ^218, 220, 222– 224, 227, 229, 232– 234^, while 34 publications report GBS due to ZIKV in 402 non travellers ^4, 44, 118, 219, 221, 225, 226, 228, 230, 231, 235– 237, 239– 247, 249, 251, 253– 260, 262, 263^.

**Study designs:** Across the different study designs, the relation between GBS and ZIKV is consistently shown. Table 2 and Extended data, Supplementary File 3 provide an overview of the included study designs ^17^.

**Lineages**: We still lack evidence on the consistency of the relation between GBS and ZIKV across different lineages from observational studies. The observed cases of GBS were linked to ZIKV of the Asian lineage.

### Risk of bias assessment

Potential selection bias in case-control studies was introduced by the selection of controls from hospitals rather than from the communities in which the cases arose ^258, 277^. Uncertainty about the exposure status due to imperfect tests would tend to result in a bias towards the null. Two case-control studies did not conduct a matched analysis although controls were matched, and no study controlled for potential confounding by factors other than those used for matching. Exclusion criteria and participation rate, especially of the controls, were poorly reported. Extended data, Supplementary File 4 provides the full risk of bias assessment of the studies included in the meta-analysis ^17^.

## Discussion

In this living systematic review, we summarised the evidence from 249 observational studies in humans on four dimensions of the causal relationship between ZIKV infection and adverse congenital outcomes and GBS, published between January 18, 2017 and July 1, 2019.

### Strengths and limitations

The strengths of this living systematic review are, first, that we automated much of the workflow ^10^; we searched both international and regional databases daily and we screen papers for eligibility as they became available, so publication bias is unlikely. Second, we have quantified the strength of association between ZIKV infection and congenital abnormalities and GBS and investigated heterogeneity of outcome and exposure assessment within and between studies. Third, for congenital outcomes, we included studies with both microcephaly and other possible adverse outcomes, acknowledging the spectrum of congenital adverse outcomes caused by ZIKV. This work also has several limitations. First, we have not assessed the dimensions of the causality framework that involve laboratory studies, so we have not updated the pathobiology of ZIKV complications, which was addressed in the baseline review ^8^ and the first update to January 2017 ^10^. Limiting the review to epidemiological domains has allowed more detailed analyses of these studies and we hope that laboratory scientists will continue to review advances in these domains. Second, the rate of publications on ZIKV remains high so, despite the reduced scope and automation, maintenance of the review is time-consuming and data extraction cannot be automated. Third, this review may suffer from continuity bias, which is important for the conduct and interpretation of living systematic reviews and results from changes in the author team. Careful adherence to the protocol will reduce this risk.

### Interpretation of the findings

ZIKV and congenital abnormalities: Since the earlier versions of the review ^8, 10^, evidence on the causal relationship between ZIKV infection and congenital abnormalities has expanded. Unfortunately, the total number of cases investigated in the published cohort or case-control studies remains small. In case-control studies in which infants with microcephaly or other congenital abnormalities are compared with unaffected infants, the strength of association differs according to whether exposure to ZIKV is assessed in in the mother (OR 3.8, 95% CI: 1.7-8.7, tau ^2^=0.18, I ^2^=19.8%) or the foetus/infant (OR 37.4, 95% CI: 11.0-127.1, tau ^2^=0, I ^2^=0%). This large difference in effect size can be attributed to the fact that not all maternal ZIKV infections result in foetal infection. In cohort studies, the risk of congenital abnormalities was 3.5 times higher (95% CI: 0.9-13.5, I ^2^=0%, tau ^2^=0) in mothers with evidence of ZIKV infection than without, which is similar to the OR for maternal exposure to ZIKV estimated from case-control studies. Further research is needed to understand the drivers of mother to child transmission. Higher maternal antibody titres were correlated with a higher incidence of adverse congenital outcomes in one case-control study ^127^. However, amongst ZIKV-infected mothers followed prospectively, severity of ZIKV infection was not associated with more severe congenital abnormalities ^136^. Convincing evidence on a dose-response relation is therefore still lacking.

ZIKV and GBS: Evidence on the causal relation between ZIKV infection and GBS has grown since our last review ^10^. The body of evidence is still smaller than that for congenital abnormalities, possibly because GBS is a rare complication, estimated to occur in 0.24 per 1000 ZIKV infections ^277^. In this review, the strength of association between GBS and ZIKV infection, estimated in case-control studies, tended to be lower than observed in the first case-control study reported by Cao-Lormeau (2016) in French Polynesia ^277^. It is possible that the finding by Cao-Lormeau *et al.* was a ‘random high’, a chance finding ^278^. Simon *et al.*, however, found a similarly strong association in a case-control study in New Caledonia ^258^. In both these studies, controls were patients in the same hospital. Although matched for place of residence, it is possible that they were less likely to have been exposed to ZIKV than the cases, resulting in an overestimation of the OR. In case-control studies in which controls were enrolled from the same communities as the cases, estimated ORs were lower, presumably because exposure to ZIKV amongst community-enrolled controls is less biased than amongst hospital controls ^279^. Under-ascertainment of ZIKV infection in case-control studies in which enrolment occurred several months after the onset of symptoms ^255, 256^ is also likely to have reduced the observed strength of association. There is also possible evidence of a dose-response relationship, with higher levels of neutralising antibodies to both ZIKV and dengue in people with GBS ^260^. However, the level of antibody titre might not be an appropriate measure of viral titre, and merely a reflection of the intensity of the immune response. Taking into account the entire body of evidence, inference to the best explanation ^280^ supports the conclusion that ZIKV is a cause of GBS. The prospect of more precise and robust estimates of the strength of association between ZIKV and GBS is low because outbreaks need to be sufficiently large to enrol enough people with GBS. In the large populations that were exposed during the 2015–2017 outbreak, herd immunity will limit future ZIKV outbreaks.

### Implications for future research

The sample sizes of studies published to date are smaller than those recommended by WHO for obtaining precise estimates of associations between ZIKV and adverse outcomes [ Harmonization of ZIKV Research Protocols to Address Key Public Health]. Given the absence of large new outbreaks of ZIKV infection in 2017–2019, there is a need for consortia of researchers to analyse their data in meta-analyses based on individual participant data [ Individual Participant Data Meta-analysis of Zika-virus related cohorts of pregnant women (ZIKV IPD-MA)]. Future collaborative efforts will help to quantify the absolute risks of different adverse congenital outcomes and allow investigation of heterogeneity between studies ^136, 149, 275^.

This review highlights additional research gaps. We did not assess the complication rates within the infected group in studies without an unexposed comparison group; the adverse outcomes are not pathognomonic for ZIKV infection, making an appropriate comparison group necessary. Although there are no individual features of ZIKV infection that are completely specific, the growing number of publications on ZIKV will allow better ascertainment of the features of a congenital Zika syndrome ^281^. In this review, we did not take into account the performance of the diagnostic tests in assessing the strength of association. Future research should include robust validation studies, and improved understanding of contextual factors in the performance of diagnostic tests, including the influence of previous circulation of other flaviviruses, the prevalence of ZIKV and the test used.

This living systematic review will continue to follow studies of adverse outcomes originating from ZIKV circulation in the Americas, but research in regions with endemic circulation of ZIKV is expected to increase. Such studies will clarify whether ZIKV circulation in Africa and Asia also results in adverse outcomes, as suggested by the case-control study of GBS from Bangladesh ^259^. Increased awareness might improve the evidence-base in these regions, where misperceptions about the potential risks of ZIKV-associated disease with different virus lineages has been reported ^282^. An important outstanding question remains whether the absence of reported cases of congenital abnormalities or GBS in these regions represent a true absence of complications or is this due to weaker surveillance systems or reporting ^283^. The conclusions that ZIKV infection causes adverse congenital outcomes and GBS are reinforced with the evidence published between January 18, 2017 and July 1, 2019.

## Data availability

### Underlying data

All data underlying the results are available as part of the article and no additional source data are required.

### Extended data

Harvard dataverse: Living systematic review on adverse outcomes of Zika - Supplementary Material. https://doi.org/10.7910/DVN/S7USUI ^17^

This project contains the following extended data:

- SupplementaryFile1Prisma.docx (PRISMA checklist)
- SupplementaryFile2Methods.docx (Supplementary file 2, additional information to the Methods)
- SupplementaryFile3Results.docx (Supplementary file 3, additional information to the Results)
- SupplementaryFile4ROB.tab (Risk of bias assessment)

Harvard dataverse: Living systematic review on adverse outcomes of Zika - Figures and Table. https://doi.org/10.7910/DVN/DLP5AN ^16^

This project contains the following extended data:

- Fig1.pdf (Most recent version of Figure 1)
- Fig2.pdf (Most recent version of Figure 3, PRISMA flowchart)
- Fig3A.pdf (Most recent version of Figure 4A)
- Fig3B.pdf (Most recent version of Figure 4B)
- Fig4.pdf (Most recent version of Figure 5)
- Fig5.pdf (Most recent version of Figure 6)
- Table1.pdf (Most recent version of Table 1)
- Table2.pdf (Most recent version of Table 2)

### Reporting guidelines

PRISMA checklist and flow diagram for ‘Zika virus infection as a cause of congenital brain abnormalities and Guillain-Barré syndrome: A living systematic review’, https://doi.org/10.7910/DVN/S7USUI and Figure 2.

## References

[1] Butler D: Drop in cases of Zika threatens large-scale trials. *Nature.* 2017;545(7655):396–7. 10.1038/545396a 28541340

[2] Colón-González FJ Peres CA Steiner São Bernardo C: After the epidemic: Zika virus projections for Latin America and the Caribbean. *PLoS Negl Trop Dis.* 2017;11(11):e0006007. 10.1371/journal.pntd.0006007 29091713 PMC5683651

[3] Hamer DH Chen LH: Zika in Angola and India. *J Travel Med.* 2019;26(5): taz012. 10.1093/jtm/taz012 30753689

[4] Umapathi T Kam YW Ohnmar O: The 2016 Singapore Zika virus outbreak did not cause a surge in Guillain-Barré syndrome. *J Peripher Nerv Syst.* 2018;23(3):197–201. 10.1111/jns.12284 30070025

[5] Ruchusatsawat K Wongjaroen P Posanacharoen A: Long-term circulation of Zika virus in Thailand: an observational study. *Lancet Infect Dis.* 2019;19(4):439–46. 10.1016/S1473-3099(18)30718-7 30826189 PMC6511259

[6] Althouse BM Vasilakis N Sall AA: Potential for Zika Virus to Establish a Sylvatic Transmission Cycle in the Americas. *PLoS Negl Trop Dis.* 2016;10(12):e0005055. 10.1371/journal.pntd.0005055 27977671 PMC5157942

[7] Heymann DL Hodgson A Sall AA: Zika virus and microcephaly: why is this situation a PHEIC? *Lancet.* 2016;387(10020):719–21. 10.1016/S0140-6736(16)00320-2 26876373 PMC7134564

[8] Krauer F Riesen M Reveiz L: Zika Virus Infection as a Cause of Congenital Brain Abnormalities and Guillain-Barré Syndrome: Systematic Review. *PLoS Med.* 2017;14(1):e1002203. 10.1371/journal.pmed.1002203 28045901 PMC5207634

[9] Elliott JH Turner T Clavisi O: Living systematic reviews: an emerging opportunity to narrow the evidence-practice gap. *PLoS Med.* 2014;11(2):e1001603. 10.1371/journal.pmed.1001603 24558353 PMC3928029

[10] Counotte M Egli-Gany D Riesen M: Zika virus infection as a cause of congenital brain abnormalities and Guillain-Barré syndrome: From systematic review to living systematic review [version 1; peer review: 2 approved, 1 approved with reservations]. *F1000Res.* 2018;7:196. 10.12688/f1000research.13704.1 30631437 PMC6290976

[11] Devakumar D Bamford A Ferreira MU: Infectious causes of microcephaly: epidemiology, pathogenesis, diagnosis, and management. *Lancet Infect Dis.* 2018;18(1):e1–e13. 10.1016/S1473-3099(17)30398-5 28844634

[12] Frenkel LD Gomez F Sabahi F: The pathogenesis of microcephaly resulting from congenital infections: why is my baby's head so small? *Eur J Clin Microbiol Infect Dis.* 2018;37(2):209–26. 10.1007/s10096-017-3111-8 28980148

[13] Koopmans M de Lamballerie X Jaenisch T: Familiar barriers still unresolved-a perspective on the Zika virus outbreak research response. *Lancet Infect Dis.* 2019;19(2):e59–e62. 10.1016/S1473-3099(18)30497-3 30420230

[14] Fischer C Pedroso C Mendrone A Jr: External Quality Assessment for Zika Virus Molecular Diagnostic Testing, Brazil. *Emerg Infect Dis.* 2018;24(5):888–892. 10.3201/eid2405.171747 29470164 PMC5938781

[15] Charrel R Mogling R Pas S: Variable Sensitivity in Molecular Detection of Zika Virus in European Expert Laboratories: External Quality Assessment, November 2016. *J Clin Microbiol.* 2017;55(11):3219–26. 10.1128/JCM.00987-17 28835479 PMC5654905

[16] Counotte MJ: Living systematic review on adverse outcomes of Zika - Figures and Table. Harvard Dataverse, V3.2019 10.7910/DVN/DLP5AN

[17] Counotte MJ: Living systematic review on adverse outcomes of Zika - Supplementary Material. Harvard Dataverse, V1, UNF:6:sAGbGceAYGoHLIT7sDtDsw== [fileUNF].2019 10.7910/DVN/S7USUI

[18] Moher D Liberati A Tetzlaff J: Preferred reporting items for systematic reviews and meta-analyses: the PRISMA statement. *PLoS Med.* 2009;6(7):e1000097. 10.1371/journal.pmed.1000097 19621072 PMC2707599

[19] Harris PA Taylor R Thielke R: Research electronic data capture (REDCap)--a metadata-driven methodology and workflow process for providing translational research informatics support. *J Biomed Inform.* 2009;42(2):377–81. 10.1016/j.jbi.2008.08.010 18929686 PMC2700030

[20] Viechtbauer W: Conducting Meta-Analyses in R with the metafor Package. *J Stat Softw.* 2010;36(3):1–48. 10.18637/jss.v036.i03

[21] Higgins JP Thompson SG: Quantifying heterogeneity in a meta-analysis. *Stat Med.* 2002;21(11):1539–58. 10.1002/sim.1186 12111919

[22] R Core Team: R: A Language and Environment for Statistical Computing. Vienna, Austria.2018 Reference Source

[23] Hernán MA Hernández-Díaz S Robins JM: A structural approach to selection bias. *Epidemiology.* 2004;15(5):615–25. 10.1097/01.ede.0000135174.63482.43 15308962

[24] Moi ML Nguyen TT Nguyen CT: Zika virus infection and microcephaly in Vietnam. *Lancet Infect Dis.* 2017;17(8):805–6. 10.1016/S1473-3099(17)30412-7 28741545

[25] Santos VS Oliveira SJG Gurgel RQ: Case Report: Microcephaly in Twins due to the Zika Virus. *Am J Trop Med Hyg.* 2017;97(1):151–4. 10.4269/ajtmh.16-1021 28719330 PMC5508915

[26] Mattar S Ojeda C Arboleda J: Case report: microcephaly associated with Zika virus infection, Colombia. *BMC Infect Dis.* 2017;17(1):423. 10.1186/s12879-017-2522-6 28610628 PMC5470308

[27] Zacharias N Whitty J Noblin S: First Neonatal Demise with Travel-Associated Zika Virus Infection in the United States of America. *AJP Rep.* 2017;7(2):e68–e73. 10.1055/s-0037-1601890 28413694 PMC5391262

[28] Benjamin I Fernández G Figueira JV: Zika virus detected in amniotic fluid and umbilical cord blood in an *in vitro* fertilization-conceived pregnancy in Venezuela. *Fertil Steril.* 2017;107(6):1319–22. 10.1016/j.fertnstert.2017.02.112 28390691

[29] Kaur G Manganas L: EEG findings in a case of congenital zika virus syndrome. *Neurology.* 2017;88(16 Supplement 1). Reference Source

[30] Saulino D Gaston E Younke B: The first zika-related infant mortality in the United States: An autopsy case report. *Laboratory Investigation.* 2017;97:10A.

[31] Zuanazzi D Arts EJ Jorge PK: Postnatal Identification of Zika Virus Peptides from Saliva. *J Dent Res.* 2017;96(10):1078–84. 10.1177/0022034517723325 28825520

[32] Lovagnini Frutos MG Ochoa JH Barbás MG: New Insights into the Natural History of Congenital Zika Virus Syndrome. *Fetal Diagn Ther.* 2018;44(1):72–6. 10.1159/000479866 28898891

[33] Villamil-Gomez WE Guijarro E Castellanos J: Congenital Zika syndrome with prolonged detection of Zika virus RNA. *J Clin Virol.* 2017;95:52–4. 10.1016/j.jcv.2017.08.010 28866135

[34] Rodó C Suy A Sulleiro E: In utero negativization of Zika virus in a foetus with serious central nervous system abnormalities. *Clin Microbiol Infect.* 2018;24(5):549 e1–e3. 10.1016/j.cmi.2017.09.022 29030170

[35] Jucá E Pessoa A Ribeiro E: Hydrocephalus associated to congenital Zika syndrome: does shunting improve clinical features? *Childs Nerv Syst.* 2018;34(1):101–6. 10.1007/s00381-017-3636-2 29086073

[36] Raymond A Jakus J: Cerebral Infarction and Refractory Seizures in a Neonate with Suspected Zika Virus Infection. *Pediatr Infect Dis J.* 2018;37(4):e112–e4. 29140935 10.1097/INF.0000000000001832

[37] Rabelo K de Souza Campos Fernandes RC de Souza LJ: Placental Histopathology and Clinical Presentation of Severe Congenital Zika Syndrome in a Human Immunodeficiency Virus-Exposed Uninfected Infant. *Front Immunol.* 2017;8:1704. 10.3389/fimmu.2017.01704 29270171 PMC5725436

[38] Angelidou A Michael Z Hotz A: Is There More to Zika? Complex Cardiac Disease in a Case of Congenital Zika Syndrome. *Neonatology.* 2018;113(2):177–82. 10.1159/000484656 29248924

[39] de Freitas Ribeiro BN Muniz BC Gasparetto EL: Congenital involvement of the central nervous system by the Zika virus in a child without microcephaly - spectrum of congenital syndrome by the Zika virus. *J Neuroradiol.* 2018;45(2):152–3. 10.1016/j.neurad.2017.11.007 29273529

[40] Chimelli L Moura Pone S Avvad-Portari E: Persistence of Zika Virus After Birth: Clinical, Virological, Neuroimaging, and Neuropathological Documentation in a 5-Month Infant With Congenital Zika Syndrome. *J Neuropathol Exp Neurol.* 2018;77(3):193–8. 10.1093/jnen/nlx116 29346650

[41] Regadas VC Silva MCE Abud LG: Microcephaly caused by congenital Zika virus infection and viral detection in maternal urine during pregnancy. *Rev Assoc Med Bras (1992).* 2018;64(1):11–4. 10.1590/1806-9282.64.01.11 29561936

[42] Schwartz KL Chan T Rai N: Zika virus infection in a pregnant Canadian traveler with congenital fetal malformations noted by ultrasonography at 14-weeks gestation. *Trop Dis Travel Med Vaccines.* 2018;4:2. 10.1186/s40794-018-0062-8 29632700 PMC5885377

[43] Prata-Barbosa A Cleto-Yamane TL Robaina JR: Co-infection with Zika and Chikungunya viruses associated with fetal death-A case report. *Int J Infect Dis.* 2018;72:25–7. 10.1016/j.ijid.2018.04.4320 29738826

[44] Rabelo K Souza LJ Salomão NG: Placental Inflammation and Fetal Injury in a Rare Zika Case Associated With Guillain-Barré Syndrome and Abortion. *Front Microbiol.* 2018;9:1018. 10.3389/fmicb.2018.01018 29867903 PMC5964188

[45] Guevara JG Agarwal-Sinha S: Ocular abnormalities in congenital Zika syndrome: a case report, and review of the literature. *J Med Case Rep.* 2018;12(1):161. 10.1186/s13256-018-1679-y 29884243 PMC5994093

[46] Giovanetti M Goes de Jesus J Lima de Maia M: Genetic evidence of Zika virus in mother's breast milk and body fluids of a newborn with severe congenital defects. *Clin Microbiol Infect.* 2018;24(10):1111–2. 10.1016/j.cmi.2018.06.008 29906587

[47] Sassetti M Zé-Zé L Franco J: First case of confirmed congenital Zika syndrome in continental Africa. *Trans R Soc Trop Med Hyg.* 2018;112(10):458–62. 10.1093/trstmh/try074 30053235

[48] Brito CAA Henriques-Souza A Soares CRP: Persistent detection of Zika virus RNA from an infant with severe microcephaly - a case report. *BMC Infect Dis.* 2018;18(1):388. 10.1186/s12879-018-3313-4 30097025 PMC6086026

[49] Gunturiz ML Cortés L Cuevas EL: Congenital cerebral toxoplasmosis, Zika and chikungunya virus infections: a case report. *Biomedica.* 2018;38(2):144–52. 10.7705/biomedica.v38i0.3652 30184357

[50] Valdespino-Vázquez MY Sevilla-Reyes EE Lira R: Congenital Zika Syndrome and Extra-Central Nervous System Detection of Zika Virus in a Pre-term Newborn in Mexico. *Clin Infect Dis.* 2019;68(6):903–12. 10.1093/cid/ciy616 30188990 PMC6399440

[51] Ventura CV Bandstra ES Fernandez MP: First Locally Acquired Congenital Zika Syndrome Case in the United States: Neonatal Clinical Manifestations. *Ophthalmic Surg Lasers Imaging Retina.* 2018;49(9):e93–e8. 10.3928/23258160-20180907-14 30222826

[52] Lemos de Carvalho A Brites C Taguchi TB: Congenital Zika Virus Infection with Normal Neurodevelopmental Outcome, Brazil. *Emerg Infect Dis.* 2018;24(11):2128–30. 10.3201/eid2411.180883 30334734 PMC6200011

[53] Ho CY Castillo N Encinales L: Second-trimester Ultrasound and Neuropathologic Findings in Congenital Zika Virus Infection. *Pediatr Infect Dis J.* 2018;37(12):1290–3. 10.1097/INF.0000000000002080 30408008 PMC6233735

[54] Wongsurawat T Jenjaroenpun P Athipanyasilp N: Genome Sequences of Zika Virus Strains Recovered from Amniotic Fluid, Placenta, and Fetal Brain of a Microcephaly Patient in Thailand, 2017. *Microbiol Resour Announc.* 2018;7(11): pii: e01020-18. 10.1128/MRA.01020-18 30533643 PMC6256666

[55] Ribeiro BNF Marchiori E: Congenital Zika syndrome associated with findings of cerebellar cortical dysplasia - Broadening the spectrum of presentation of the syndrome. *J Neuroradiol.* 2018; pii: S0150-9861(18)30261-X. 10.1016/j.neurad.2018.10.009 30448428

[56] Lockrow J Tully H Saneto RP: Epileptic spasms as the presenting seizure type in a patient with a new "O" of TORCH, congenital Zika virus infection. *Epilepsy Behav Case Rep.* 2018;11:1–3. 10.1016/j.ebcr.2018.09.002 30456170 PMC6232624

[57] Davila-Castrodad NM Reyes-Bou Z Correa-Rivas M: First Autopsy of a Newborn with Congenital Zika Syndrome in Puerto Rico. *P R Health Sci J.* 2018;37(Special Issue):S81–S4. 30576583

[58] Yarrington CD Hamer DH Kuohung W: Congenital Zika syndrome arising from sexual transmission of Zika virus, a case report. *Fertil Res Pract.* 2019;5: 1. 10.1186/s40738-018-0053-5 30619616 PMC6317256

[59] Khatib A Showler AJ Kain D: A diagnostic gap illuminated by a sexually-transmitted case of congenital Zika virus infection. *Travel Med Infect Dis.* 2019;27:117–8. 10.1016/j.tmaid.2018.10.017 30739645

[60] Santos GR Pinto CAL Prudente RCS: Case Report: Histopathologic Changes in Placental Tissue Associated With Vertical Transmission of Zika Virus. *Int J Gynecol Pathol.* 2019. 10.1097/PGP.0000000000000586 30789499

[61] Sulleiro E Frick MA Rodó C: The challenge of the laboratory diagnosis in a confirmed congenital Zika virus syndrome in utero: A case report. *Medicine (Baltimore).* 2019;98(20):e15532. 10.1097/MD.0000000000015532 31096455 PMC6531038

[62] de Paula Freitas B Zin A Ko A: Anterior-Segment Ocular Findings and Microphthalmia in Congenital Zika Syndrome. *Ophthalmology.* 2017;124(12):1876–8. 10.1016/j.ophtha.2017.06.009 28676282

[63] Linden VV Linden HV Junior Leal MC: Discordant clinical outcomes of congenital Zika virus infection in twin pregnancies. *Arq Neuropsiquiatr.* 2017;75(6):381–6. 10.1590/0004-282X20170066 28658408

[64] Leal MC van der Linden V Bezerra TP: Characteristics of Dysphagia in Infants with Microcephaly Caused by Congenital Zika Virus Infection, Brazil, 2015. *Emerg Infect Dis.* 2017;23(8):1253–9. 10.3201/eid2308.170354 28604336 PMC5547788

[65] Parra-Saavedra M Reefhuis J Piraquive JP: Serial Head and Brain Imaging of 17 Fetuses With Confirmed Zika Virus Infection in Colombia, South America. *Obstet Gynecol.* 2017;130(1):207–12. 10.1097/AOG.0000000000002105 28594771 PMC5511628

[66] Aragao MFVV Holanda AC Brainer-Lima AM: Nonmicrocephalic Infants with Congenital Zika Syndrome Suspected Only after Neuroimaging Evaluation Compared with Those with Microcephaly at Birth and Postnatally: How Large Is the Zika Virus "Iceberg"? *AJNR Am J Neuroradiol.* 2017;38(7):1427–34. 10.3174/ajnr.A5216 28522665 PMC7959892

[67] Cabral CM Nóbrega M Leite PLE: Clinical-epidemiological description of live births with microcephaly in the state of Sergipe, Brazil, 2015. *Epidemiol Serv Saude.* 2017;26(2):245–54. 10.5123/S1679-49742017000200002 28492766

[68] Sousa AQ Cavalcante DIM Franco LM: Postmortem Findings for 7 Neonates with Congenital Zika Virus Infection. *Emerg Infect Dis.* 2017;23(7):1164–7. 10.3201/eid2307.162019 28459414 PMC5512501

[69] Ventura LO Ventura CV Lawrence L: Visual impairment in children with congenital Zika syndrome. *J AAPOS.* 2017;21(4):295–299.e2. 10.1016/j.jaapos.2017.04.003 28450178

[70] Ramalho FS Yamamoto AY da Silva LL: Congenital Zika Virus Infection Induces Severe Spinal Cord Injury. *Clin Infect Dis.* 2017;65(4):687–90. 10.1093/cid/cix374 28444144

[71] Cavalcanti DD Alves LV Furtado GJ: Echocardiographic findings in infants with presumed congenital Zika syndrome: Retrospective case series study. *PLoS One.* 2017;12(4):e0175065. 10.1371/journal.pone.0175065 28426680 PMC5398518

[72] Yepez JB Murati FA Pettito M: Ophthalmic Manifestations of Congenital Zika Syndrome in Colombia and Venezuela. *JAMA Ophthalmol.* 2017;135(5):440–5. 10.1001/jamaophthalmol.2017.0561 28418539 PMC5470423

[73] Aragao MFVV Brainer-Lima AM Holanda AC: Spectrum of Spinal Cord, Spinal Root, and Brain MRI Abnormalities in Congenital Zika Syndrome with and without Arthrogryposis. *AJNR Am J Neuroradiol.* 2017;38(5):1045–53. 10.3174/ajnr.A5125 28364011 PMC7960394

[74] Chimelli L Melo ASO Avvad-Portari E: The spectrum of neuropathological changes associated with congenital Zika virus infection. *Acta Neuropathol.* 2017;133(6):983–99. 10.1007/s00401-017-1699-5 28332092

[75] Meneses JDA Ishigami AC de Mello LM: Lessons Learned at the Epicenter of Brazil's Congenital Zika Epidemic: Evidence From 87 Confirmed Cases. *Clin Infect Dis.* 2017;64(10):1302–8. 10.1093/cid/cix166 28329257

[76] Del Campo M Feitosa IM Ribeiro EM: The phenotypic spectrum of congenital Zika syndrome. *Am J Med Genet A.* 2017;173(4):841–57. 10.1002/ajmg.a.38170 28328129

[77] Acosta-Reyes J Navarro E Herrera MJ: Severe Neurologic Disorders in 2 Fetuses with Zika Virus Infection, Colombia. *Emerg Infect Dis.* 2017;23(6):982–4. 10.3201/eid2306.161702 28296632 PMC5443437

[78] Sanín-Blair JE Gutiérrez-Márquez C Herrera DA: Fetal Magnetic Resonance Imaging Findings in Prenatal Zika Virus Infection. *Fetal Diagn Ther.* 2017;42(2):153–7. 10.1159/000454860 28288452

[79] Schaub B Vouga M Najioullah F: Analysis of blood from Zika virus-infected fetuses: a prospective case series. *Lancet Infect Dis.* 2017;17(5):520–7. 10.1016/S1473-3099(17)30102-0 28209336

[80] Tse C Picon M Rodriguez P: The Effects of Zika in Pregnancy: The Miami Experience [20M]. *Obstet Gynecol.* 2017;129:137s-8s 10.1097/01.AOG.0000514691.21416.35

[81] Aleman TS Ventura CV Cavalcanti MM: Quantitative Assessment of Microstructural Changes of the Retina in Infants With Congenital Zika Syndrome. *JAMA Ophthalmol.* 2017;135(10):1069–76. 10.1001/jamaophthalmol.2017.3292 28880978 PMC5710497

[82] Mejdoubi M Monthieux A Cassan T: Brain MRI in Infants after Maternal Zika Virus Infection during Pregnancy. *N Engl J Med.* 2017;377(14):1399–400. 10.1056/NEJMc1612813 28976853

[83] Fernandez MP Parra Saad E Ospina Martinez M: Ocular Histopathologic Features of Congenital Zika Syndrome. *JAMA Ophthalmol.* 2017;135(11):1163–9. 10.1001/jamaophthalmol.2017.3595 28975230 PMC5710450

[84] Petribu NCL Aragao MFV van der Linden V: Follow-up brain imaging of 37 children with congenital Zika syndrome: case series study. *BMJ.* 2017;359:j4188. 10.1136/bmj.j4188 29030384 PMC5639438

[85] Schaub B Gueneret M Jolivet E: Ultrasound imaging for identification of cerebral damage in congenital Zika virus syndrome: a case series. *Lancet Child Adolesc Health.* 2017;1(1):45–55. 10.1016/S2352-4642(17)30001-9 30169227

[86] James-Powell T Brown Y Christie CDC: Trends of Microcephaly and Severe Arthrogryposis in Three Urban Hospitals following the Zika, Chikungunya and Dengue Fever Epidemics of 2016 in Jamaica. *West Indian Med J.* 2017;66(1):10–9. 10.7727/wimj.2017.124

[87] Contreras-Capetillo SN Valadéz-González N Manrique-Saide P: Birth Defects Associated With Congenital Zika Virus Infection in Mexico. *Clin Pediatr (Phila).* 2018;57(8):927–36. 10.1177/0009922817738341 29152996

[88] Castro JDV Pereira LP Dias DA: Presumed Zika virus-related congenital brain malformations: the spectrum of CT and MRI findings in fetuses and newborns. *Arq Neuropsiquiatr.* 2017;75(10):703–10. 10.1590/0004-282X20170134 29166461

[89] Mulkey SB Vezina G Bulas DI: Neuroimaging Findings in Normocephalic Newborns With Intrauterine Zika Virus Exposure. *Pediatr Neurol.* 2018;78:75–8. 10.1016/j.pediatrneurol.2017.10.012 29167058

[90] Pires P Jungmann P Galvão JM: Neuroimaging findings associated with congenital Zika virus syndrome: case series at the time of first epidemic outbreak in Pernambuco State, Brazil. *Childs Nerv Syst.* 2018;34(5):957–63. 10.1007/s00381-017-3682-9 29209885

[91] Felix A Hallet E Favre A: Cerebral injuries associated with Zika virus in utero exposure in children without birth defects in French Guiana: Case report. *Medicine (Baltimore).* 2017;96(51):e9178. 10.1097/MD.0000000000009178 29390455 PMC5758157

[92] Ramos CL Moreno-Carvalho OA Nascimento-Carvalho CM: Cerebrospinal fluid aspects of neonates with or without microcephaly born to mothers with gestational Zika virus clinical symptoms. *J Infect.* 2018;76(6):563–9. 10.1016/j.jinf.2018.02.004 29432825

[93] Soares-Marangoni DA Tedesco NM Nascimento AL: General movements and motor outcomes in two infants exposed to Zika virus: brief report. *Dev Neurorehabil.* 2019;22(1):71–4. 10.1080/17518423.2018.1437843 29452026

[94] Howard A Visintine J Fergie J: Two Infants with Presumed Congenital Zika Syndrome, Brownsville, Texas, USA, 2016-2017. *Emerg Infect Dis.* 2018;24(4):625–30. 10.3201/eid2404.171545 29553331 PMC5875277

[95] De Fatima Viana Vasco Aragao M Van Der Linden V Petribu NC: Congenital Zika Syndrome: Comparison of brain CT scan with postmortem histological sections from the same subjects. *Neuroradiology.* 2018;60(1 Supplement 1):367–8.

[96] Rosenstierne MW Schaltz-Buchholzer F Bruzadelli F: Zika Virus IgG in Infants with Microcephaly, Guinea-Bissau, 2016. *Emerg Infect Dis.* 2018;24(5):948–50. 10.3201/eid2405.180153 29664391 PMC5938792

[97] Oliveira-Filho J Felzemburgh R Costa F: Seizures as a Complication of Congenital Zika Syndrome in Early Infancy. *Am J Trop Med Hyg.* 2018;98(6):1860–2. 10.4269/ajtmh.17-1020 29692307 PMC6086187

[98] Walker CL Merriam AA Ohuma EO: Femur-sparing pattern of abnormal fetal growth in pregnant women from New York City after maternal Zika virus infection. *Am J Obstet Gynecol.* 2018;219(2):187.e1–187.e20. 10.1016/j.ajog.2018.04.047 29738748 PMC6066422

[99] Peixoto Filho AAA de Freitas SB Ciosaki MM: Computed tomography and magnetic resonance imaging findings in infants with microcephaly potentially related to congenital Zika virus infection. *Radiol Bras.* 2018;51(2):119–22. 10.1590/0100-3984.2016.0135 29743741 PMC5935408

[100] De Alcantara T La Beaud AD Aronoff D: CT Scan Findings in Microcephaly Cases During 2015–2016 Zika Outbreak: A Cohort Study. *Neurology.* 2018;90(15 Supplement 1). Reference Source

[101] Cardoso TF Jr Santos RSD Corrêa RM: Congenital Zika infection: neurology can occur without microcephaly. *Arch Dis Child.* 2019;104(2):199–200. 10.1136/archdischild-2018-314782 29858269

[102] Alves LV Mello MJG Bezerra PG: Congenital Zika Syndrome and Infantile Spasms: Case Series Study. *J Child Neurol.* 2018;33(10):664–6. 10.1177/0883073818780105 29897010

[103] De Moraes CG Pettito M Yepez JB: Optic neuropathy and congenital glaucoma associated with probable Zika virus infection in Venezuelan patients. *JMM Case Rep.* 2018;5(5):e005145. 10.1099/jmmcr.0.005145 29896405 PMC5994708

[104] Figueredo LF Franco-Zuluaga JA Carrere-Rivera C: Anatomical Findings of fetuses Vertically-infected with Zika Virus. *FASEB J.* 2018;32(1). Reference Source

[105] De Alcantara T Da Silva Maia JT Aronoff D: Radiological findings and neurological disorders in microcephaly cases related to zika virus: A cohort study. *Neurology.* 2018;90(15 Supplement 1). Reference Source

[106] Almeida IMLM Ramos CV Rodrigues DC: Clinical and epidemiological aspects of microcephaly in the state of Piauí, northeastern Brazil, 2015-2016. *J Pediatr (Rio J).* 2019;95(4):466–474. 10.1016/j.jped.2018.04.013 29963988

[107] Wongsurawat T Athipanyasilp N Jenjaroenpun P: Case of Microcephaly after Congenital Infection with Asian Lineage Zika Virus, Thailand. *Emerg Infect Dis.* 2018;24(9):1758–1761. 10.3201/eid2409.180416 29985788 PMC6106416

[108] Rajapakse NS Ellsworth K Liesman RM: Unilateral Phrenic Nerve Palsy in Infants with Congenital Zika Syndrome. *Emerg Infect Dis.* 2018;24(8):1422–1427. 10.3201/eid2408.180057 30016248 PMC6056128

[109] Beaufrere A Bessieres B Bonniere M: A clinical and histopathological study of malformations observed in fetuses infected by the Zika virus. *Brain Pathol.* 2019;29(1):114–125. 10.1111/bpa.12644 30020561 PMC8028325

[110] Chu V Petersen LR Moore CA: Possible Congenital Zika Syndrome in Older Children Due to Earlier Circulation of Zika Virus. *Am J Med Genet A.* 2018;176(9):1882–9. 10.1002/ajmg.a.40378 30070773

[111] Vouga M Baud D Jolivet E: Congenital Zika virus syndrome...what else? Two case reports of severe combined fetal pathologies. *BMC Pregnancy Childbirth.* 2018;18(1):356. 10.1186/s12884-018-1998-4 30176812 PMC6122623

[112] de Noronha L Zanluca C Burger M: Zika Virus Infection at Different Pregnancy Stages: Anatomopathological Findings, Target Cells and Viral Persistence in Placental Tissues. *Front Microbiol.* 2018;9:2266. 10.3389/fmicb.2018.02266 30337910 PMC6180237

[113] Martins RS Froes MH Saad LDC: Descriptive report of cases of congenital syndrome associated with Zika virus infection in the state of São Paulo, Brazil, from 2015 to 2017. *Epidemiol Serv Saude.* 2018;27(3):e2017382. 10.5123/S1679-49742018000300012 30365699

[114] Rodriguez J Diaz M Montalvo A: Case Series: Congenital Zika Virus Infection Associated with Epileptic Spasms. *Ann Neurol.* 2018;84(Supplement 22):S401–S. 10.1002/ana.25305

[115] Azevedo RSS Araujo MT Oliveira CS: Zika Virus Epidemic in Brazil. II. Post-Mortem Analyses of Neonates with Microcephaly, Stillbirths, and Miscarriage. *J Clin Med.* 2018;7(12): pii: E496. 10.3390/jcm7120496 30487475 PMC6306831

[116] Petribu NCL Fernandes ACV Abath MB: Common findings on head computed tomography in neonates with confirmed congenital Zika syndrome. *Radiol Bras.* 2018;51(6):366–71. 10.1590/0100-3984.2017.0119 30559553 PMC6290748

[117] Seferovic MD Turley M Valentine GC: Clinical Importance of Placental Testing among Suspected Cases of Congenital Zika Syndrome. *Int J Mol Sci.* 2019;20(3): pii: E712. 10.3390/ijms20030712 30736425 PMC6387308

[118] Cano M Esquivel R: Zika virus infection in Hospital del Niño 'Dr José Renán Esquivel' (Panamá): Case Review since the introduction in Latin America. *Pediátr Panamá.* 2018;47(3):15–9. Reference Source

[119] de Fatima Viana Vasco Aragao M van der Linden V Petribu NC: Congenital Zika Syndrome: The Main Cause of Death and Correspondence Between Brain CT and Postmortem Histological Section Findings From the Same Individuals. *Top Magn Reson Imaging.* 2019;28(1):29–33. 10.1097/RMR.0000000000000194 30817678

[120] Santana MB Lamas CC Athayde JG: Congenital Zika syndrome: is the heart part of its spectrum? *Clin Microbiol Infect.* 2019;25(8):1043–4. 10.1016/j.cmi.2019.03.020 30922930

[121] Venturi G Fortuna C Alves RM: Epidemiological and clinical suspicion of congenital Zika virus infection: Serological findings in mothers and children from Brazil. *J Med Virol.* 2019;91(9):1577–1583. 10.1002/jmv.25504 31090222 PMC6773202

[122] Lee EH Cooper H Iwamoto M: First 12 Months of Life for Infants in New York City, New York, With Possible Congenital Zika Virus Exposure. *J Pediatric Infect Dis Soc.* 2019; pii: piz027. 10.1093/jpids/piz027 31125410 PMC7358042

[123] Merriam AA Nhan-Chang CL Huerta-Bogdan BI: A Single-Center Experience with a Pregnant Immigrant Population and Zika Virus Serologic Screening in New York City. *Am J Perinatol.* 2019. 10.1055/s-0039-1688819 31146294

[124] Zambrano H Waggoner J Leon K: High incidence of Zika virus infection detected in plasma and cervical cytology specimens from pregnant women in Guayaquil, Ecuador. *Am J Reprod Immunol.* 2017;77(2):e12630. 10.1111/aji.12630 28177195

[125] Santa Rita TH Barra RB Peixoto GP: Association between suspected Zika virus disease during pregnancy and giving birth to a newborn with congenital microcephaly: a matched case-control study. *BMC Res Notes.* 2017;10(1):457. 10.1186/s13104-017-2796-1 28877754 PMC5588708

[126] de Araujo TVB Ximenes RAA Miranda-Filho DB: Association between microcephaly, Zika virus infection, and other risk factors in Brazil: final report of a case-control study. *Lancet Infect Dis.* 2018;18(3):328–36. 10.1016/S1473-3099(17)30727-2 29242091 PMC7617036

[127] Moreira-Soto A Sarno M Pedroso C: Evidence for Congenital Zika Virus Infection From Neutralizing Antibody Titers in Maternal Sera, Northeastern Brazil. *J Infect Dis.* 2017;216(12):1501–4. 10.1093/infdis/jix539 29272526 PMC5853373

[128] Krow-Lucal ER de Andrade MR Cananéa JNA Moore CA: Association and birth prevalence of microcephaly attributable to Zika virus infection among infants in Paraíba Brazil, in 201 6: a case-control study. *The Lancet Child & Adolescent Health.* 2018;2(3):205–13. 10.1016/S2352-4642(18)30020-8 30169255

[129] Ventura LO Ventura CV Dias NC: Visual impairment evaluation in 119 children with congenital Zika syndrome. *J AAPOS.* 2018;22(3):218–22 e1. 10.1016/j.jaapos.2018.01.009 29654909 PMC12286703

[130] Moreira-Soto A Cabral R Pedroso C: Exhaustive TORCH Pathogen Diagnostics Corroborate Zika Virus Etiology of Congenital Malformations in Northeastern Brazil. *mSphere.* 2018;3(4): pii: e00278-18. 10.1128/mSphere.00278-18 30089647 PMC6083096

[131] Subissi L Dub T Besnard M: Zika Virus Infection during Pregnancy and Effects on Early Childhood Development, French Polynesia, 2013-2016. *Emerg Infect Dis.* 2018;24(10):1850–8. 10.3201/eid2410.172079 30226164 PMC6154169

[132] Lima GP Rozenbaum D Pimentel C: Factors associated with the development of Congenital Zika Syndrome: a case-control study. *BMC Infect Dis.* 2019;19(1):277. 10.1186/s12879-019-3908-4 30902046 PMC6431070

[133] Pedroso C Fischer C Feldmann M: Cross-Protection of Dengue Virus Infection against Congenital Zika Syndrome, Northeastern Brazil. *Emerg Infect Dis.* 2019;25(8):1485–1493. 10.3201/eid2508.190113 31075077 PMC6649334

[134] Zin AA Tsui I Rossetto J: Screening Criteria for Ophthalmic Manifestations of Congenital Zika Virus Infection. *JAMA Pediatr.* 2017;171(9):847–54. 10.1001/jamapediatrics.2017.1474 28715527 PMC5710409

[135] Vercosa I Carneiro P Vercosa R: The visual system in infants with microcephaly related to presumed congenital Zika syndrome. *J AAPOS.* 2017;21(4):300–4 e1. 10.1016/j.jaapos.2017.05.024 28652051

[136] Halai UA Nielsen-Saines K Moreira ML: Maternal Zika Virus Disease Severity, Virus Load, Prior Dengue Antibodies, and Their Relationship to Birth Outcomes. *Clin Infect Dis.* 2017;65(6):877–83. 10.1093/cid/cix472 28535184 PMC5848222

[137] Reynolds MR Jones AM Petersen EE: Vital Signs: Update on Zika Virus-Associated Birth Defects and Evaluation of All U.S. Infants with Congenital Zika Virus Exposure - U.S. Zika Pregnancy Registry, 2016. *MMWR Morb Mortal Wkly Rep.* 2017;66(13):366–73. 10.15585/mmwr.mm6613e1 28384133 PMC5657905

[138] Adhikari EH Nelson DB Johnson KA: Infant outcomes among women with Zika virus infection during pregnancy: results of a large prenatal Zika screening program. *Am J Obstet Gynecol.* 2017;216(3):292 e1–e8. 10.1016/j.ajog.2017.01.018 28153665

[139] Spiliopoulos D Wooton G Economides DL: Surveillance of pregnant women exposed to Zika virus areas. *Bjog-Int J Obstet Gy.* 2017;124:17–49. 10.1111/1471-0528.14586

[140] Eppes C Rac M Dempster C: Zika Virus in a Non-Endemic Urban Population: Patient Characteristics and Ultrasound Findings. *Obstet Gynecol.* 2017;129:135s-s 10.1097/01.AOG.0000514683.06169.08

[141] Sohan K Cyrus CA: Ultrasonographic observations of the fetal brain in the first 100 pregnant women with Zika virus infection in Trinidad and Tobago. *Int J Gynaecol Obstet.* 2017;139(3):278–83. 10.1002/ijgo.12313 28842988

[142] Kam YW Leite JA Lum FM: Specific Biomarkers Associated With Neurological Complications and Congenital Central Nervous System Abnormalities From Zika Virus-Infected Patients in Brazil. *J Infect Dis.* 2017;216(2):172–81. 10.1093/infdis/jix261 28838147 PMC5853428

[143] Ximenes ASFC Pires P Werner H: Neuroimaging findings using transfontanellar ultrasound in newborns with microcephaly: a possible association with congenital Zika virus infection. *J Matern Fetal Neonatal Med.* 2019;32(3):493–501. 10.1080/14767058.2017.1384459 28942698

[144] Terzian ACB Estofolete CF Alves da Silva R: Long-Term Viruria in Zika Virus-Infected Pregnant Women, Brazil, 2016. *Emerg Infect Dis.* 2017;23(11):1891–3. 10.3201/eid2311.170078 29048293 PMC5652423

[145] Nogueira ML Nery Junior NRR Estofolete CF: Adverse birth outcomes associated with Zika virus exposure during pregnancy in São José do Rio Preto, Brazil. *Clin Microbiol Infect.* 2018;24(6):646–52. 10.1016/j.cmi.2017.11.004 29133154

[146] Sanz Cortes M Rivera AM Yepez M: Clinical assessment and brain findings in a cohort of mothers, fetuses and infants infected with ZIKA virus. *Am J Obstet Gynecol.* 2018;218(4):440 e1–e36. 10.1016/j.ajog.2018.01.012 29353032

[147] Maykin M Avaad-Portari E Esquivel M: Placental Histopathologic Findings in Zika-infected Pregnancies. *Am J Obstet Gynecol.* 2018;218(1):S520–S1. 10.1016/j.ajog.2017.11.411

[148] Adhikari EH McKiever M Nelson DB: Neonatal surveillance among asymptomatic Zika-exposed infants through 6 months of life. *Am J Obstet Gynecol.* 2018;218(1):S512–S3. 10.1016/j.ajog.2017.11.397

[149] Hoen B Schaub B Funk AL: Pregnancy Outcomes after ZIKV Infection in French Territories in the Americas. *N Engl J Med.* 2018;378(11):985–94. 10.1056/NEJMoa1709481 29539287

[150] Collier ARY Barouch DH: Maternal Immune Response to Zika Virus. *Reproductive Sciences.* 2018;25(1):163a-4a.

[151] Bulas D Mulkey S Vezina G: Fetal and postnatal brain imaging for the detection of ZIKV encephalopathy in the fetus/newborn. *Pediatric Radiology.* 2018;48(1 Supplement 1):S141.

[152] Rodriguez-Morales AJ Cardona-Ospina JA Ramirez-Jaramillo V: Diagnosis and outcomes of pregnant women with Zika virus infection in two municipalities of Risaralda, Colombia: Second report of the ZIKERNCOL study. *Travel Med Infect Dis.* 2018;25:20–5. 10.1016/j.tmaid.2018.06.006 29894797

[153] Rice ME Galang RR Roth NM: *Vital Signs*: Zika-Associated Birth Defects and Neurodevelopmental Abnormalities Possibly Associated with Congenital Zika Virus Infection - U.S. Territories and Freely Associated States, 2018. *MMWR Morb Mortal Wkly Rep.* 2018;67(31):858–67. 10.15585/mmwr.mm6731e1 30091967 PMC6089332

[154] Tsui I Moreira MEL Rossetto JD: Eye Findings in Infants With Suspected or Confirmed Antenatal Zika Virus Exposure. *Pediatrics.* 2018;142(4): pii: e20181104. 10.1542/peds.2018-1104 30213843 PMC6317824

[155] Dávila-Camargo A Durán-Nah JJ Dupinet-Sánchez A: Ophthalmologic findings in newborns of women with Zika virus infection during pregnancy: A case series. *Revista Mexicana de Oftalmología.*(English Edition)2018;92(4):159–62. 10.24875/RMOE.M18000001

[156] Pomar L Vouga M Lambert V: Maternal-fetal transmission and adverse perinatal outcomes in pregnant women infected with Zika virus: prospective cohort study in French Guiana. *BMJ.* 2018;363:k4431. 10.1136/bmj.k4431 30381296 PMC6207920

[157] Brock M Magalhaes AQ Di Tommaso Leao J: Zika virus infection in pregnant women in Manaus. *Int J Gynaecol Obstet.* 2018;143(Supplement 3):725–6. 10.1002/ijgo.12583

[158] Caixeta R Magalhaes AC De Brito WI: Vertical transmission of zika virus and perinatal microcephaly. *Int J Gynaecol Obstet.* 2018;143(Supplement 3):725 10.1002/ijgo.12583

[159] Rosado L Gomes MBF Lacerda FA: Cohort of infected Zika Virus pregnant women from central Brazil: Preliminary report. *Int J Gynaecol Obstet.* 2018;143(Supplement 3):228 10.1002/ijgo.12582

[160] Mulkey SB Bulas DI Vezina G: Sequential Neuroimaging of the Fetus and Newborn With In Utero Zika Virus Exposure. *JAMA Pediatr.* 2019;173(1):52–9. 10.1001/jamapediatrics.2018.4138 30476967 PMC6583436

[161] Gely-Rojas L Pérez R García-Fragoso L: Association of Zika Virus Exposure in Utero with Ocular Phenotypes in a Group of Newborns in Puerto Rico Exposure with ocular phenotypes in newborns in Puerto Rico. *P R Health Sci J.* 2018;37(Special Issue):S77–S80. 30576582

[162] Gely-Rojas L García-Fragoso L Negrón J: Congenital Zika Syndrome in Puerto Rico, Beyond Microcephaly, A Multiorgan Approach. *P R Health Sci J.* 2018;37(Special Issue):S73–S6. 30576581

[163] Pereira JP Jr Maykin MM Vasconcelos Z: The Role of Amniocentesis in the Diagnosis of Congenital Zika Syndrome. *Clin Infect Dis.* 2019;69(4):713–716. 10.1093/cid/ciz013 30624579 PMC6669287

[164] Pereira JP Jr Nielsen-Saines K Sperling J: Association of Prenatal Ultrasonographic Findings With Adverse Neonatal Outcomes Among Pregnant Women With Zika Virus Infection in Brazil. *JAMA Netw Open.* 2018;1(8):e186529. 10.1001/jamanetworkopen.2018.6529 30646333 PMC6324324

[165] Del Carpio-Orantes L Rosas-Lozano AL García-Méndez S: Zika virus infection in pregnant women in a General Hospital of Veracruz, Mexico. *J Matern Fetal Neonatal Med.* 2019;1–5. 10.1080/14767058.2019.1582627 30760071

[166] Rodó C Suy A Sulleiro E: Pregnancy outcomes after maternal Zika virus infection in a non-endemic region: prospective cohort study. *Clin Microbiol Infect.* 2019;25(5):633.e5–633.e9. 10.1016/j.cmi.2019.02.008 30771526

[167] Calle-Giraldo JP Rojas CA Hurtado IC: Outcomes of Congenital Zika Virus Infection During an Outbreak in Valle del Cauca, Colombia. *Pediatr Infect Dis J.* 2019;38(7):735–40. 10.1097/INF.0000000000002307 30985517

[168] Baran LCP da Costa MF Vidal KS: Alterations in visual acuity and visual development in infants 1-24 months old either exposed to or infected by Zika virus during gestation, with and without microcephaly. *J AAPOS.* 2019; pii: S1091-8531(19)30137-5. 10.1016/j.jaapos.2019.03.005 31229606

[169] de Magalhães-Barbosa MC Prata-Barbosa A Robaina JR: Prevalence of microcephaly in eight south-eastern and midwestern Brazilian neonatal intensive care units: 2011-2015. *Arch Dis Child.* 2017;102(8):728–34. 10.1136/archdischild-2016-311541 28302630

[170] Merriam AA Nhan-Chang CL Huerta-Bogdan BI: 393: A single-center experience with a pregnant immigrant population and zika virus in new york city. *Am J Perinatol.* 2017;216(1):S236-S. 10.1016/j.ajog.2016.11.651 31146294

[171] Gregianini TS Ranieri T Favreto C: Emerging arboviruses in Rio Grande do Sul, Brazil: Chikungunya and Zika outbreaks, 2014-2016. *Rev Med Virol.* 2017;27(6). 10.1002/rmv.1943 28929534

[172] Netto EM Moreira-Soto A Pedroso C: High Zika Virus Seroprevalence in Salvador, Northeastern Brazil Limits the Potential for Further Outbreaks. *MBio.* 2017;8(6): pii: e01390-17. 10.1128/mBio.01390-17 29138300 PMC5686533

[173] Shiu C Starker R Kwal J: Zika Virus Testing and Outcomes during Pregnancy, Florida, USA, 2016. *Emerg Infect Dis.* 2018;24(1):1–8. 10.3201/eid2401.170979 29260671 PMC5749464

[174] Mletzko J Schildgen O: Absence of Zika virus in abortion and placental tissue in a German cohort. *Rev Med Microbiol.* 2018;29(1):17–9. Reference Source

[175] Marques Abramov D Saad T Gomes-Junior SC: Auditory brainstem function in microcephaly related to Zika virus infection. *Neurology.* 2018;90(7):e606–e14. 10.1212/WNL.0000000000004974 29352094

[176] Orofino DHG Passos SRL de Oliveira RVC: Cardiac findings in infants with in utero exposure to Zika virus- a cross sectional study. *PLoS Negl Trop Dis.* 2018;12(3):e0006362. 10.1371/journal.pntd.0006362 29579059 PMC5886696

[177] Fonteles CSR Marques Ribeiro E Sales Aragão Santos M: Lingual Frenulum Phenotypes in Brazilian Infants With Congenital Zika Syndrome. *Cleft Palate Craniofac J.* 2018;55(10):1391–8. 10.1177/1055665618766999 29613837

[178] Ferreira HNC Schiariti V Regalado ICR: Functioning and Disability Profile of Children with Microcephaly Associated with Congenital Zika Virus Infection. *Int J Environ Res Public Health.* 2018;15(6): pii: E1107. 10.3390/ijerph15061107 29844290 PMC6025082

[179] Da Maia JTS Vidal SJA Mendes TV: Perinatal Case Fatality Rate in Cases of Congenital Zika Syndrome: a Cross-Sectional Study. *Neurology.* 2018;90(15 Supplement 1). Reference Source

[180] João EC Ferreira ODC Jr Gouvêa MI: Pregnant women co-infected with HIV and Zika: Outcomes and birth defects in infants according to maternal symptomatology. *PLoS One.* 2018;13(7):e0200168. 10.1371/journal.pone.0200168 29979796 PMC6034846

[181] Herber S Silva AA Sanseverino MTV: Prevalence and causes of congenital microcephaly in the absence of a Zika virus outbreak in southern Brazil. *J Pediatr (Rio J).* 2018; pii: S0021-7557(18)30119-0. 10.1016/j.jped.2018.05.013 31340900

[182] Zin AA Tsui I Rossetto JD: Visual function in infants with antenatal Zika virus exposure. *J AAPOS.* 2018;22(6):452–456.e1. 10.1016/j.jaapos.2018.07.352 30359768 PMC6289819

[183] Kikuti M Cardoso CW Prates APB: Congenital brain abnormalities during a Zika virus epidemic in Salvador, Brazil, April 2015 to July 2016. *Euro Surveill.* 2018;23(45): 1700757. 10.2807/1560-7917.ES.2018.23.45.1700757 30424827 PMC6234531

[184] Alayed MS Qureshi MA Ahmed S: Seroprevalence of Zika virus among asymptomatic pregnant mothers and their newborns in the Najran region of southwest Saudi Arabia. *Ann Saudi Med.* 2018;38(6):408–12. 10.5144/0256-4947.2018.408 30531174 PMC6302992

[185] Peña F Pimentel R Khosla S: Zika Virus Epidemic in Pregnant Women, Dominican Republic, 2016-2017. *Emerg Infect Dis.* 2019;25(2):247–55. 10.3201/eid2502.181054 30666928 PMC6346438

[186] C Lage ML Carvalho AL Ventura PA: Clinical, Neuroimaging, and Neurophysiological Findings in Children with Microcephaly Related to Congenital Zika Virus Infection. *Int J Environ Res Public Health.* 2019;16(3): pii: E309. 10.3390/ijerph16030309 30678125 PMC6388186

[187] Carvalho FR Medeiros T Vianna RAO: Simultaneous circulation of arboviruses and other congenital infections in pregnant women in Rio de Janeiro, Brazil. *Acta Trop.* 2019;192(NA):49–54. 10.1016/j.actatropica.2019.01.020 30685232

[188] Jaenisch T Rosenberger KD Brito C: Risk of microcephaly after Zika virus infection in Brazil, 2015 to 2016. *Bull World Health Organ.* 2017;95(3):191–8. 10.2471/BLT.16.178608 28250532 PMC5328112

[189] Souza WV Albuquerque MFPM Vazquez E: Microcephaly epidemic related to the Zika virus and living conditions in Recife, Northeast Brazil. *BMC Public Health.* 2018;18(1): 130. 10.1186/s12889-018-5039-z 29329574 PMC5767029

[190] Vissoci JRN Rocha TAH Silva NCD: Zika virus infection and microcephaly: Evidence regarding geospatial associations. *PLoS Negl Trop Dis.* 2018;12(4):e0006392. 10.1371/journal.pntd.0006392 29694351 PMC5937996

[191] Souza AI de Siqueira MT Ferreira ALCG: Geography of Microcephaly in the Zika Era: A Study of Newborn Distribution and Socio-environmental Indicators in Recife, Brazil, 2015-2016. *Public Health Rep.* 2018;133(4):461–71. 10.1177/0033354918777256 29920225 PMC6055288

[192] Majumder MS Hess R Ross R: Seasonality of birth defects in West Africa: could congenital Zika syndrome be to blame? [version 2; peer review: 2 approved, 1 approved with reservations] *F1000Res.* 2018;7:159. 10.12688/f1000research.13858.2 30079237 PMC6058464

[193] Campos MC Dombrowski JG Phelan J: Zika might not be acting alone: Using an ecological study approach to investigate potential co-acting risk factors for an unusual pattern of microcephaly in Brazil. *PLoS One.* 2018;13(8):e0201452. 10.1371/journal.pone.0201452 30110370 PMC6093667

[194] Brady OJ Osgood-Zimmerman A Kassebaum NJ: The association between Zika virus infection and microcephaly in Brazil 2015-2017: An observational analysis of over 4 million births. *PLoS Med.* 2019;16(3):e1002755. 10.1371/journal.pmed.1002755 30835728 PMC6400331

[195] Bautista LE: Maternal Zika virus infection and newborn microcephaly-an analysis of the epidemiological evidence. *Ann Epidemiol.* 2018;28(2):111–8. 10.1016/j.annepidem.2017.11.010 29277550

[196] Hernández-Ávila JE Palacio-Mejía LS López-Gatell H: Zika virus infection estimates, Mexico. *Bull World Health Organ.* 2018;96(5):306–13. 10.2471/BLT.17.201004 29875515 PMC5985421

[197] Hay JA Nouvellet P Donnelly CA: Potential inconsistencies in Zika surveillance data and our understanding of risk during pregnancy. *PLoS Negl Trop Dis.* 2018;12(12):e0006991. 10.1371/journal.pntd.0006991 30532143 PMC6301717

[198] Hurtado-Villa P Puerto AK Victoria S: Raised Frequency of Microcephaly Related to Zika Virus Infection in Two Birth Defects Surveillance Systems in Bogotá and Cali, Colombia. *Pediatr Infect Dis J.* 2017;36(10):1017–9. 10.1097/INF.0000000000001670 28914749

[199] de Oliveira WK de França GVA Carmo EH: Infection-related microcephaly after the 2015 and 2016 Zika virus outbreaks in Brazil: a surveillance-based analysis. *Lancet.* 2017;390(10097):861–70. 10.1016/S0140-6736(17)31368-5 28647172

[200] Shapiro-Mendoza CK Rice ME Galang RR: Pregnancy Outcomes After Maternal Zika Virus Infection During Pregnancy - U.S. Territories, January 1, 2016-April 25, 2017. *MMWR Morb Mortal Wkly Rep.* 2017;66(23):615–21. 10.15585/mmwr.mm6623e1 28617773 PMC5657842

[201] de Oliveira WK Carmo EH Henriques CM: Zika Virus Infection and Associated Neurologic Disorders in Brazil. *N Engl J Med.* 2017;376(16):1591–3. 10.1056/NEJMc1608612 28402236 PMC5544116

[202] Magalhães-Barbosa MC Prata-Barbosa A Robaina JR: New trends of the microcephaly and Zika virus outbreak in Brazil, July 2016-December 2016. *Travel Med Infect Dis.* 2017;16:52–7. 10.1016/j.tmaid.2017.03.009 28342826

[203] Journel I Andrécy LL Metellus D: Transmission of Zika Virus - Haiti, October 12, 2015-September 10, 2016. *MMWR Morb Mortal Wkly Rep.* 2017;66(6):172–6. 10.15585/mmwr.mm6606a4 28207688 PMC5657860

[204] Neto NNM Da Silva Maia JT Zacarkim MR: Mortality in newborns with microcephaly due to maternal zika virus infection in Rio Grande Do Norte state-northeast Brazil: A crosssectional study. *Neurology.* 2017;88(16 Supplement 1). Reference Source

[205] Hall NB Broussard K Evert N: Notes from the Field: Zika Virus-Associated Neonatal Birth Defects Surveillance - Texas, January 2016-July 2017. *MMWR Morb Mortal Wkly Rep.* 2017;66(31):835–6. 10.15585/mmwr.mm6631a5 28796762 PMC5687783

[206] Méndez N Oviedo-Pastrana M Mattar S: Zika virus disease, microcephaly and Guillain-Barré syndrome in Colombia: epidemiological situation during 21 months of the Zika virus outbreak, 2015-2017. *Arch Public Health.* 2017;75:65. 10.1186/s13690-017-0233-5 29118981 PMC5667031

[207] Delaney A Mai C Smoots A: Population-Based Surveillance of Birth Defects Potentially Related to Zika Virus Infection - 15 States and U.S. Territories, 2016. *MMWR Morb Mortal Wkly Rep.* 2018;67(3):91–6. 10.15585/mmwr.mm6703a2 29370151 PMC5812309

[208] Ribeiro IG Andrade MR Silva JM: Microcephaly in Piauí, Brazil: descriptive study during the Zika virus epidemic, 2015-2016. *Epidemiol Serv Saude.* 2018;27(1):e20163692. 10.5123/S1679-49742018000100002 29412347

[209] Mendes Neto NN da Silva Maia JT Zacarkim MR: Perinatal Case Fatality Rate Related to Congenital Zika Syndrome in Brazil: A Cross-Sectional Study. *Pediatr Neurol.* 2018;81:47–8. 10.1016/j.pediatrneurol.2017.11.012 29526344

[210] Sowunmi S Eckert V Griffin A: 58 ^th^ Annual Teratology Society Meeting. *Birth Defects Res.* 2018;110(9):667–822. 10.1002/bdr2.1355 29878724

[211] Tellechea AL Luppo V Morales MA: Surveillance of microcephaly and selected brain anomalies in Argentina: Relationship with Zika virus and other congenital infections. *Birth Defects Res.* 2018;110(12):1016–26. 10.1002/bdr2.1347 29921033

[212] França GVA Pedi VD Garcia MHO: Congenital syndrome associated with Zika virus infection among live births in Brazil: a description of the distribution of reported and confirmed cases in 2015-2016. *Epidemiol Serv Saude.* 2018;27(2):e2017473. 10.5123/S1679-49742018000200014 29972474

[213] Porse CC Messenger S Vugia DJ: Travel-Associated Zika Cases and Threat of Local Transmission during Global Outbreak, California, USA. *Emerg Infect Dis.* 2018;24(9):1626–32. 10.3201/eid2409.180203 30124194 PMC6106427

[214] Silva JHD Terças ACP Pinheiro LCB: Profile of congenital anomalies among live births in the municipality of Tangará da Serra, Mato Grosso, Brazil, 2006-2016. *Epidemiol Serv Saude.* 2018;27(3):e2018008. 10.5123/S1679-49742018000300017 30365695

[215] Spiliopoulos D Panchal M Economides DL: Surveillance of pregnant women with potential exposure to Zika virus following travel. *G Chir.* 2019;40(1):58–65. 30771801

[216] Fernández Martínez B Martínez Sánchez EV Diaz Garcia O: Zika virus disease in Spain. Surveillance results and epidemiology on reported cases, 2015-2017. *Med Clin (Barc).* 2019;153(1):6–12. 10.1016/j.medcli.2018.12.014 30797578

[217] Sulleiro E Rando A Alejo I: Screening for Zika virus infection in 1057 potentially exposed pregnant women, Catalonia (northeastern Spain). *Travel Med Infect Dis.* 2019;29:69–71. 10.1016/j.tmaid.2019.03.006 30885699

[218] Miller E Becker Z Shalev D: Probable Zika virus-associated Guillain-Barré syndrome: Challenges with clinico-laboratory diagnosis. *J Neurol Sci.* 2017;375:367–70. 10.1016/j.jns.2017.02.029 28320169

[219] Raboni SM Bonfim C Almeida BM: Flavivirus cross-reactivity in serological tests and Guillain-Barré syndrome in a hematopoietic stem cell transplant patient: A case report. *Transpl Infect Dis.* 2017;19(4). 10.1111/tid.12700 28306183

[220] Tantillo G Sclar G Vasa C: Zika associated Guillain-Barre syndrome in the United States. *Neurology.* 2017;88(16 Supplement 1). Reference Source

[221] Santos P Nogueira F Modenezi L: Movement disorder development later after acute disseminated encephalomyelitis associated with Zika virus: Case report. *Neurology.* 2017;88(16 Supplement 1). Reference Source

[222] Beattie J Parajuli S Sanger M: Zika Virus-Associated Guillain-Barre Syndrome In A Returning United States Traveler. *Am J Respir Crit Care Med.* 2017;195:A2008 Reference Source

[223] Wu Y Cui X Wu N: A unique case of human Zika virus infection in association with severe liver injury and coagulation disorders. *Sci Rep.* 2017;7(1):11393. 10.1038/s41598-017-11568-4 28900143 PMC5595821

[224] Leonhard SE Munts AG van der Eijk AA: Acute-onset chronic inflammatory demyelinating polyneuropathy after Zika virus infection. *J Neurol Neurosurg Psychiatry.* 2017;89(10):1118–1119. 10.1136/jnnp-2017-317346 29175895

[225] Dirlikov E Torres JV Martines RB: Postmortem Findings in Patient with Guillain-Barré Syndrome and Zika Virus Infection. *Emerg Infect Dis.* 2018;24(1):114–7. 10.3201/eid2401.171331 29261094 PMC5749436

[226] Siwarit R: Guillain-Barre syndrome in the Context of Zika Virus Infection: the First Report Case in Thailand.2017;34(2). Reference Source

[227] Gibelin N Romero J Gregoire V: Miller Fisher syndrome associated with a Zika virus infection. *Eur J Neurol.* 2018;25(2):e20–e1. 10.1111/ene.13514 29356259

[228] Gonzalez-Escobar G Valadere AM Adams R: Prolonged Zika virus viremia in a patient with Guillain-Barré syndrome in Trinidad and Tobago. *Rev Panam Salud Publica.* 2018;41:e136. 10.26633/RPSP.2017.136 29466521 PMC6645378

[229] Jones M: Multidisciplinary approach to Guillain-Barre Syndrome and neurologic injuries after zika virus. *J Spinal Cord Med.* 2018;41(5):588.

[230] Mancera-Paez O Román GC Pardo-Turriago R: Concurrent Guillain-Barré syndrome, transverse myelitis and encephalitis post-Zika: A case report and review of the pathogenic role of multiple arboviral immunity. *J Neurol Sci.* 2018;395:47–53. 10.1016/j.jns.2018.09.028 30292020

[231] Cordeiro de Souza L de Souza AA de Almeida EEP: Inspiratory Muscle Training with Isokinetic Device to Help Ventilatory Weaning in a Patient with Guillain-Barré Syndrome by Zika Virus. *Case Rep Crit Care.* 2018;2018:9708451. 10.1155/2018/9708451 30402297 PMC6193329

[232] Rivera-Concepción JR Betancourt JP Cerra J: The Zika Virus: An Association to Guillain-Barré Syndrome in the United States - A Case Report. *P R Health Sci J.* 2018;37(Spec Issue):S93–S5. 30576585

[233] Wright JK Castellani L Lecce C: Zika Virus-Associated Aseptic Meningitis and Guillain-Barre Syndrome in a Traveler Returning from Latin America: a Case Report and Mini-Review. *Curr Infect Dis Rep.* 2019;21(1):3. 10.1007/s11908-019-0661-1 30767073

[234] Boggild AK Geduld J Libman M: Surveillance report of Zika virus among Canadian travellers returning from the Americas. *CMAJ.* 2017;189(9):E334–E40. 10.1503/cmaj.161241 28280063 PMC5334005

[235] Villamil-Gomez WE Sánchez-Herrera ÁR Hernandez H: Guillain-Barré syndrome during the Zika virus outbreak in Sucre, Colombia, 2016. *Travel Med Infect Dis.* 2017;16:62–3. 10.1016/j.tmaid.2017.03.012 28347781

[236] Ali A Williams M: Zik-V outbreak and Guillain-Barre syndrome in Jamaica. *Neurology.* 2017;88(16 Supplement 1). Reference Source

[237] Francois R Berkowitz A: Zika-associated atypical guillain-barre variants in rural haiti. *Neurology.* 2017;88(16 Supplement 1). Reference Source

[238] Barreira AA Marques W Marreco A: Guilllain-barre syndrome related to Zika virus infection in Brazil. *Neurology.* 2017;88(16 Supplement 1). Reference Source

[239] Betances N Luciano C Carlo J: Prominent distal demyelination is a feature of zika-associated guillain barre syndrome. *Neurology.* 2017;88(16 Supplement 1). Reference Source

[240] Sebastián UU Ricardo AVA Alvarez BC: Zika virus-induced neurological critical illness in Latin America: Severe Guillain-Barre Syndrome and encephalitis. *J Crit Care.* 2017;42:275–81. 10.1016/j.jcrc.2017.07.038 Reference Source 28806562 PMC7127615

[241] da Silva IRF Frontera JA Bispo de Filippis AM: Neurologic Complications Associated With the Zika Virus in Brazilian Adults. *JAMA Neurol.* 2017;74(10):1190–8. 10.1001/jamaneurol.2017.1703 28806453 PMC5710239

[242] Del Carpio Orantes L Juárez Rangel FJ García-Méndez S: Incidence of Guillain-Barré syndrome at a secondary centre during the 2016 zika outbreak. *Neurologia.* 2017; pii: S0213-4853(17)30279-7. 10.1016/j.nrl.2017.07.019 28958396

[243] Dourado ME Fernandes U Vital AL: High Incidence of Guillain-Barre Syndrome after Zika Virus Infection in the State Rio Grande Do Norte, in Northeast Brazil. *J Peripher Nerv Syst.* 2017;22(3):275–6.

[244] Roze B Najioullah F Ferge JL: Guillain-Barré Syndrome Associated With Zika Virus Infection in Martinique in 2016: A Prospective Study. *Clin Infect Dis.* 2017;65(9):1462–8. 10.1093/cid/cix588 29020245

[245] Brito Ferreira ML Antunes de Brito CA Moreira ÁJP: Guillain-Barré Syndrome, Acute Disseminated Encephalomyelitis and Encephalitis Associated with Zika Virus Infection in Brazil: Detection of Viral RNA and Isolation of Virus during Late Infection. *Am J Trop Med Hyg.* 2017;97(5):1405–9. 10.4269/ajtmh.17-0106 29140242 PMC5817749

[246] Resiere D Ferge JL Fergé J: Cardiovascular complications in patients with Zika virus-induced Guillain-Barré syndrome. *J Clin Virol.* 2018;98:8–9. 10.1016/j.jcv.2017.11.002 29175232

[247] Uncini A Gonzalez-Bravo DC Acosta-Ampudia YY: Clinical and nerve conduction features in Guillain-Barré syndrome associated with Zika virus infection in Cúcuta, Colombia. *Eur J Neurol.* 2018;25(4):644–50. 10.1111/ene.13552 29266602

[248] Mehta R Soares CN Medialdea-Carrera R: The spectrum of neurological disease associated with Zika and chikungunya viruses in adults in Rio de Janeiro, Brazil: A case series. *PLoS Negl Trop Dis.* 2018;12(2):e0006212. 10.1371/journal.pntd.0006212 29432457 PMC5837186

[249] Dirlikov E Major CG Medina NA: Clinical Features of Guillain-Barré Syndrome With vs Without Zika Virus Infection, Puerto Rico, 2016. *JAMA Neurol.* 2018;75(9):1089–97. 10.1001/jamaneurol.2018.1058 29799940 PMC6143122

[250] Azevedo MB Coutinho MSC Silva MAD: Neurologic manifestations in emerging arboviral diseases in Rio de Janeiro City, Brazil, 2015-2016. *Rev Soc Bras Med Trop.* 2018;51(3):347–51. 10.1590/0037-8682-0327-2017 29972566

[251] Nóbrega MEBD Araujo ELL Wada MY: Outbreak of Guillain-Barré syndrome possibly related to prior Zika virus infection, Metropolitan Region of Recife, Pernambuco, Brazil, 2015. *Epidemiol Serv Saude.* 2018;27(2):e2017039. 10.5123/S1679-49742018000200016 29995102

[252] Baskar D Amalnath D Mandal J: Antibodies to Zika virus, *Campylobacter jejuni* and gangliosides in Guillain-Barre syndrome: A prospective single-center study from southern India. *Neurol India.* 2018;66(5):1324–31. 10.4103/0028-3886.241402 30232998

[253] Zambrano LI Fuentes-Barahona IC Soto-Fernandez RJ: Guillain-Barré syndrome associated with Zika virus infection in Honduras, 2016-2017. *Int J Infect Dis.* 2019;84:136–7. 10.1016/j.ijid.2019.05.008 31096053

[254] Soto-Hernandez JL Ponce de Leon Rosales S Vargas Canas ES: Guillain-Barré Syndrome Associated With Zika Virus Infection: A Prospective Case Series From Mexico. *Front Neurol.* 2019;10:435. 10.3389/fneur.2019.00435 31114537 PMC6502985

[255] Styczynski AR Malta JMAS Krow-Lucal ER: Increased rates of Guillain-Barré syndrome associated with Zika virus outbreak in the Salvador metropolitan area, Brazil. *PLoS Negl Trop Dis.* 2017;11(8):e0005869. 10.1371/journal.pntd.0005869 28854206 PMC5595339

[256] Salinas JL Walteros DM Styczynski A: Zika virus disease-associated Guillain-Barré syndrome-Barranquilla, Colombia 2015-2016. *J Neurol Sci.* 2017;381:272–7. 10.1016/j.jns.2017.09.001 28991697

[257] Dirlikov E Medina NA Major CG: Acute Zika Virus Infection as a Risk Factor for Guillain-Barré Syndrome in Puerto Rico. *JAMA.* 2017;318(15):1498–500. 10.1001/jama.2017.11483 29049645 PMC5817969

[258] Simon O Acket B Forfait C: Zika virus outbreak in New Caledonia and Guillain-Barré syndrome: a case-control study. *J Neurovirol.* 2018;24(3):362–8. 10.1007/s13365-018-0621-9 29594985

[259] GeurtsvanKessel CH Islam Z Islam MB: Zika virus and Guillain-Barré syndrome in Bangladesh. *Ann Clin Transl Neurol.* 2018;5(5):606–15. 10.1002/acn3.556 29761123 PMC5945960

[260] Lynch RM Mantus G Encinales L: Augmented Zika and Dengue Neutralizing Antibodies Are Associated With Guillain-Barré Syndrome. *J Infect Dis.* 2019;219(1):26–30. 10.1093/infdis/jiy466 30113672 PMC6284544

[261] Munoz LS Barréras P Lizarazo J: Neuroviruses Emerging in the Americas Study(NEAS): The Colombian experience during the 2016 outbreak of Zika virus infection. *Neurology.* 2017;88(16 Supplement 1). Reference Source

[262] Brito KGDS Dos Santos EB Lucas LDSM: Prevalence of neurological complications associated with Zika virus in a brazilian metropolis. *Neurol Int.* 2018;10(2):7638. 10.4081/ni.2018.7638 30069289 PMC6050448

[263] Del Carpio-Orantes L Peniche Moguel KG Sanchez Diaz JS: Guillain-Barré syndrome associated with Zika virus infection: Analysis of a cohort from the region of northern Veracruz in 2016-2017. *Neurologia.* 2018; pii: S0213-4853(18)30173-7. 10.1016/j.nrl.2018.05.002 30072275

[264] Ikejezie J Shapiro CN Kim J: Zika Virus Transmission - Region of the Americas, May 15, 2015-December 15, 2016. *MMWR Morb Mortal Wkly Rep.* 2017;66(12):329–34. 10.15585/mmwr.mm6612a4 28358795 PMC5657956

[265] Mier-Y-Teran-Romero L Delorey MJ Sejvar JJ: Guillain-Barré syndrome risk among individuals infected with Zika virus: a multi-country assessment. *BMC Med.* 2018;16(1):67. 10.1186/s12916-018-1052-4 29759069 PMC5952697

[266] Vieira MADCES Cruz ACR Barros ANM: *Guillain-Barré* syndrome and dengue-like disease in 2015: temporal relationship in *Piauí* state and implications on Zika virus surveillance. *Rev Inst Med Trop Sao Paulo.* 2017;59:e22. 10.1590/S1678-9946201759022 28423097 PMC5441001

[267] Malta JM Vargas A Leite PL: Guillain-Barré syndrome and other neurological manifestations possibly related to Zika virus infection in municipalities from Bahia, Brazil, 2015. *Epidemiol Serv Saude.* 2017;26(1):9–18. 10.5123/S1679-49742017000100002 28226004

[268] Tolosa N Tinker SC Pacheco O: Zika Virus Disease in Children in Colombia, August 2015 to May 2016. *Paediatr Perinat Epidemiol.* 2017;31(6):537–45. 10.1111/ppe.12391 28806479

[269] Brenciaglia M Noel TP Fields PJ: Clinical, Serological, and Molecular Observations from a Case Series Study during the Asian Lineage Zika Virus Outbreak in Grenada during 2016. *Can J Infect Dis Med Microbiol.* 2018;2018:4635647. 10.1155/2018/4635647 29623138 PMC5829423

[270] Vieira MADCES Costa CHN Linhares ADC: Potential role of dengue virus, chikungunya virus and Zika virus in neurological diseases. *Mem Inst Oswaldo Cruz.* 2018;113(11):e170538. 10.1590/0074-02760170538 30379197 PMC6204615

[271] de Araujo TVB Rodrigues LC de Alencar Ximenes RA: Association between Zika virus infection and microcephaly in Brazil, January to May, 2016: preliminary report of a case-control study. *Lancet Infect Dis.* 2016;16(12):1356–63. 10.1016/S1473-3099(16)30318-8 27641777 PMC7617035

[272] Barreras P Pamies D Kumar A: 2016 Annual Meetings. *Ann Neurol.* 2016;80(s20):S1–S432. 10.1002/ana.24759 27739587 PMC7159757

[273] Pomar L Malinger G Benoist G: Association between Zika virus and fetopathy: a prospective cohort study in French Guiana. *Ultrasound Obstet Gynecol.* 2017;49(6):729–36. 10.1002/uog.17404 28078779

[274] Brasil P Pereira JP Jr Moreira ME: Zika Virus Infection in Pregnant Women in Rio de Janeiro. *N Engl J Med.* 2016;375(24):2321–34. 10.1056/NEJMoa1602412 26943629 PMC5323261

[275] Honein MA Dawson AL Petersen EE: Birth Defects Among Fetuses and Infants of US Women With Evidence of Possible Zika Virus Infection During Pregnancy. *JAMA.* 2017;317(1):59–68. 10.1001/jama.2016.19006 27960197

[276] Costa Monteiro LM Cruz GNO Fontes JM: Neurogenic bladder findings in patients with Congenital Zika Syndrome: A novel condition. *PLoS One.* 2018;13(3):e0193514. 10.1371/journal.pone.0193514 29494684 PMC5832242

[277] Cao-Lormeau VM Blake A Mons S: Guillain-Barré Syndrome outbreak associated with Zika virus infection in French Polynesia: a case-control study. *Lancet.* 2016;387(10027):1531–9. 10.1016/S0140-6736(16)00562-6 26948433 PMC5444521

[278] Vandenbroucke JP: Case reports in an evidence-based world. *J R Soc Med.* 1999;92(4):159–63. 10.1177/014107689909200401 10450190 PMC1297135

[279] Kopec JA Esdaile JM: Bias in case-control studies. A review. *J Epidemiol Community Health.* 1990;44(3):179–86. 10.1136/jech.44.3.179 2273353 PMC1060638

[280] Lipton P: Inference to the best explanation. Routledge; 2003. Reference Source

[281] Moore CA Staples JE Dobyns WB: Characterizing the Pattern of Anomalies in Congenital Zika Syndrome for Pediatric Clinicians. *JAMA Pediatr.* 2017;171(3):288–95. 10.1001/jamapediatrics.2016.3982 27812690 PMC5561417

[282] Grubaugh ND Ishtiaq F Setoh YX: Misperceived Risks of Zika-related Microcephaly in India. *Trends Microbiol.* 2019;27(5):381–383. 10.1016/j.tim.2019.02.004 30826180

[283] Nutt C Adams P: Zika in Africa-the invisible epidemic? *Lancet.* 2017;389(10079):1595–6. 10.1016/S0140-6736(17)31051-6 28593839

