# Supplementary material for: Zika virus infection as a cause of congenital brain abnormalities and Guillain-Barré syndrome: A living systematic review
Source: F1000Res. 2019 Aug 14;8:1433. [Version 1] doi: 10.12688/f1000research.19918.1 (PMC6852328; doi:10.12688/f1000research.19918.1)
Supplement: Peer review history

# Peer review: Reviewer response for version 1

**DOI:** 10.5256/f1000research.21857.r55660

**Published:** 2019-11-12

**Authors:** Ana B.G. Veiga

**Affiliations:** 1 Federal University of Health Sciences of Porto Alegre (UFCSPA), Porto Alegre, Brazil

The article is a well-written systematic review of studies that show that Zika virus infection is associated with congenital abnormalities, and with Guillain-Barré syndrome.

 Because it is a living systematic review, the study gives interesting and important updates.

 There are a few grammar errors that must be corrected:

- In Methods, correct is "This review and subsequent updates will focus on four..."
- In the sentence "Araujo et al. found a 73.1 (95% CI 13·0–Inf) times higher odds was reported for microcephaly...", I would delete "was reported", so it would be better if written "Araujo et al. found a 73.1 (95% CI 13·0–Inf) times higher odds for microcephaly when ZIKV infection was assessed by reverse transcription polymerase chain reaction (RT-PCR) in the neonate." (page 6)
- PRNT (plaque reduction neutralisation test) should be defined when first mentioned.
- Correct non traveller to non-traveller (pages 9 and 10).

The text mentions Figure 4 as showing the combined adverse congenital outcomes, and Figure 5 as showing microcephaly as outcome. However, this is not clear in the figures. In addition, why are the diagnostic testes considered outcomes in Figure 5? 

 Figures should be self-explanatory, so I suggest mentioning GBS in the legend of Figure 6 so that the reader doesn't need to refer to the text. Also, VNT should be specified as virus neutralisation test in the figure legend.

I confirm that I have read this submission and believe that I have an appropriate level of expertise to confirm that it is of an acceptable scientific standard.

# Peer review: Reviewer response for version 1

**DOI:** 10.5256/f1000research.21857.r53510

**Published:** 2019-10-18

**Authors:** Rajesh Verma

**Affiliations:** 1 Department of Neurology, King George's Medical University, Lucknow, Uttar Pradesh, India

I think this is an excellent review written on the topic Zika virus and adverse fetal outcome and neurological complication (Guillain Barre Syndrome).

 The studies interpretation in terms of statistical significance is outstanding.

 The pathogenesis of these complications are not mentioned. How GBS develops after Zika infection? Whether immune mediated or virus has direct affinity for nervous system?

 What precautions to be taken at community level to curb the menace of Zika virus infection?

I confirm that I have read this submission and believe that I have an appropriate level of expertise to confirm that it is of an acceptable scientific standard.
